# An interpretable framework for sleep posture change detection and postural inactivity segmentation using wrist kinematics

**DOI:** 10.1038/s41598-023-44567-9

**Published:** 2023-10-21

**Authors:** Omar Elnaggar, Roselina Arelhi, Frans Coenen, Andrew Hopkinson, Lyndon Mason, Paolo Paoletti

**Affiliations:** 1 School of Engineering, University of Liverpool, Liverpool, L69 3GH UK; 2 Faculty of Engineering, University of Sheffield, Sheffield, S1 3JD UK; 3 School of Electrical Engineering, Electronics and Computer Science, University of Liverpool, Liverpool, L69 3BX UK; 4 School of Psychology, University of Liverpool, Liverpool, L69 7ZA UK; 5 School of Medicine, University of Liverpool, Liverpool, L69 3GE UK; 6 Department of Trauma and Orthopaedics, Liverpool University Hospitals NHS Foundation Trust, Liverpool, L9 7AL UK

**Keywords:** Engineering, Applied mathematics

## Abstract

Sleep posture and movements offer insights into neurophysiological health and correlate with overall well-being and quality of life. Clinical practices utilise polysomnography for sleep assessment, which is intrusive, performed in unfamiliar environments, and requires trained personnel. While sensor technologies such as actigraphy are less invasive alternatives, concerns about their reliability and precision in clinical practice persist. Moreover, the field lacks a universally accepted algorithm, with methods ranging from raw signal thresholding to data-intensive classification models that may be unfamiliar to medical staff. This paper proposes a comprehensive framework for objectively detecting sleep posture changes and temporally segmenting postural inactivity using clinically relevant joint kinematics, measured by a custom-made wearable sensor. The framework was evaluated on wrist kinematic data from five healthy participants during simulated sleep. Intuitive three-dimensional visualisations of kinematic time series were achieved through dimension reduction-based preprocessing, providing an out-of-the-box framework explainability that may be useful for clinical monitoring and diagnosis. The proposed framework achieved up to 99.2% F1-score and 0.96 Pearson’s correlation coefficient for posture detection and inactivity segmentation respectively. This work paves the way for reliable home-based sleep movement analysis, serving patient-centred longitudinal care.

## Introduction

The study of human sleep behaviour reveals their state of health and well-being. Habitual in-bed behaviour can reveal physiological and neurological disorders that are otherwise latent during wakefulness^1^ such as *restless leg syndrome* and *periodic leg movements*. Sleep deprivation and intermittent sleep were found to be linked to multiple health risks^2–5^. In-bed sleep behaviour (movements and postures) could cause health complications, such as *pressure sores*^6^, apnoea^7^ and painful spasms^8,9^.

**Figure 1:**
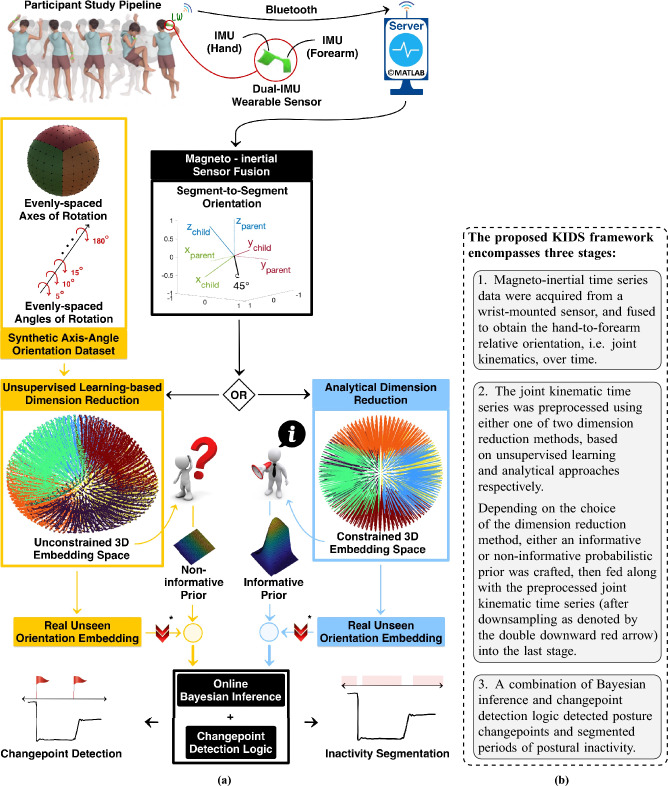
Graphical illustration (**a**) with explanatory text (**b**) of the proposed kinematics-based (in)activity detection and segmentation (KIDS) framework, a novel framework for wearable sensor-based sleep posture change detection and temporal segmentation of postural inactivity during sleep.

In light of the clinical context outlined above, there has been a growing interest within the research community to study human sleep behaviour. Different aspects were investigated including sleep posture classification^10,11^, detection of in-bed movements and posture transitions^12,13^, sleep staging^14^, sleep physiology and vital sign monitoring^15,16^. Various technologies were employed for at-home and in-clinic sleep monitoring. The clinical gold standard for the assessment of sleep-related disorders has been *polysomnography* (PSG) which measures multiple physiological parameters. There are, however, disadvantages to using PSG such as sensor and electrode intrusiveness, unfamiliar sleep environment, and cost of personnel training and technology. Therefore, alternatives to PSG were proposed to make less sophisticated sleep assessments. Popular options included the less intrusive *accelerometer-based sensing* (actigraphy^17^) which involved an actigraphic device, such as a smartwatch worn around the wrist or ankle, to record motor activity during sleep and measure parameters like sleep quality and duration. Other solutions adopted *bed-embodied sensors*, such as load cells^12^, and *in-bedroom sensors* such as app-empowered smartphones^18^ which incorporated multiple sensors like accelerometers and microphones.

Within the large field of in-bed movement analysis, there were commonly three research directions reported in the literature: *active/idle state detection*, *wake/sleep state detection* and *sleep stage estimation*. From the literature, these directions broadly relied on similar methodologies, namely threshold-based, classification-based and hybrid approaches. Threshold-based approaches^12,17,19–25^ were the most popular, applying a predefined threshold hyperparameter to a predictor variable (raw data or processed features) to classify sensor time series on a sample or window basis. Classification-based approaches^18,20,21,26^ employed classifiers to recognise states, such as the active and idle states, based on sensor measurements or features. The less popular hybrid approaches^17,27^ used a mixture of threshold- and classification-based approaches; a thresholding algorithm typically produced preliminary labels which were then refined by a classifier to improve performance. The previous approaches had shortcomings, such as the detection of short-lasting wake state surrounded by long-lasting sleep state^28^. Therefore, it was common in the literature to employ handcrafted “*re-scoring rules*” to correct such systematic errors^29^. Nevertheless, this set of rules needed to be applied with caution as they might favour accuracy over $F_1$-score or even degrade both of them^28^. Additional insights can be found in comparative studies^28,30^ which analysed the performance of previous approaches with a focus on actigraphy.

The limitations of existing work on body movement analysis during sleep can therefore be summarised as follows. First, while reported approaches predominantly addressed various forms of state detection problems on a sample or window basis, they overlooked the importance of temporal analysis for time series data. Second, threshold-based approaches heavily relied on tricky-to-tweak hyperparameters, which lack generalisability given that different subjects exhibit varying in-bed behaviour and movement intensities. Third, classification-based approaches required large-size datasets for classifier training, and these datasets were typically imbalanced in nature (disproportionate class-wise sample size) and lacked diversity among participants^28^. Fourth, the reported approaches generally operated on raw sensor data or manually extracted features which are not necessarily the best representation of information for movement analysis, nor best comprehensible to medical experts.

This paper proposes a novel *kinematics-based (in)activity detection and segmentation* (KIDS) framework (depicted in Fig. 1). Leveraging the kinematics of a single body joint, the KIDS framework jointly addressed two interrelated problems: sleep posture change detection and temporal segmentation of postural inactivity. The effectiveness of the framework was demonstrated through a pilot study involving five human participants, each wearing a miniature sensor module embedded with two inertial measurement units (IMUs) around their left wrist joint.

The choice of exploiting joint kinematics was motivated by the authors’ recent research which indicated the efficacy of similar kinematic cues from extremity joints (wrists and ankles) in identifying 12 sleep postures^11^. However, this previous work required manual segmentation of each posture to showcase the posture classification performance. Herein lies an example of the potential application of the KIDS framework, with its ability to automate cumbersome tasks, such as temporal segmentation, which have often been manually performed by human experts. Broadly, the KIDS framework serves as a milestone towards automated and non-intrusive assessment of human postural (in)activity during sleep, thereby paving the way for the next generation of diagnostic and treatment practices in sleep medicine.

The primary contributions of the presented work can be summarised as follows:

1. We propose the first utilisation of *magneto-inertial sensor fusion* in the domain of temporal analysis of in-bed postural activity. Our framework evaluated the whole-body physical activity level by examining a single joint’s segment-to-segment orientation (also referred to as joint kinematics), as demonstrated on the wrist joint. Compared to conventional methods that rely on raw inertial sensor measurements, joint kinematics is better suited for human interpretability and medical diagnosis. Additionally, the framework eliminated the need for sensor-to-segment calibration before implementation, making it more accessible to non-expert users.
2. We propose and demonstrate the practical application of dimension reduction techniques as a preprocessing step for joint kinematics. This step served to produce intuitive three-dimensional visualisations of joint kinematic time series while simultaneously mitigating the computational burden of the algorithm. The dimension reduction process was achieved by employing two distinct methods: an established unsupervised learning approach and a novel analytical approximation of the former. It is important to note that these methods were mutually exclusive, meaning only one of them was utilised at a time rather than in combination.
3. We propose a novel framework that integrated *Bayesian inference* with *changepoint detection logic* to jointly address sleep posture change detection and temporal segmentation of postural inactivity. Notably, the approach distinguished itself by its capacity to function autonomously, without relying on intricate parameters that require meticulous fine-tuning and lack generalisability, a common issue with threshold-based algorithms. Moreover, the need for collecting and labelling training data was circumvented since the framework did not involve trainable classifiers.
4. We publish an open data repository containing the preprocessed joint kinematics from all five human participants who partook in an ethically approved simulated sleep experimental protocol devised to evaluate the effectiveness of the KIDS framework. This repository is available to researchers and practitioners, allowing them to access and use the data to drive further advancements in the field of temporal analysis of in-bed postural activity.

## Results

The proposed KIDS framework, shown in Fig. 1, involved three stages: (1) wrist kinematics measurement using wearable inertial sensors, (2) joint kinematics preprocessing and visualisation through dimension reduction methods, and (3) sleep posture change detection and temporal segmentation of postural inactivity using Bayesian inference and changepoint detection logic.

The KIDS framework had four variants resulting from methodological choices made during its development. The first crucial decision arose at the preprocessing stage of measured joint kinematics, which involved the utilisation of one of two dimension reduction methods. The second decision was related to the incorporation of an optional enhancement step prior to the changepoint detection logic. As a consequence, each crossroad contributed two possible variants, ultimately yielding a total of four variants.

The comparison between the four variants would entail elaborating methodological aspects. Hence, “Results” section will only present the best-performing variant of the KIDS framework, providing key highlights from each of its three stages. An aggregated analysis of all four variants will follow in “Methods” section, where the methodological foundation underpinning the proposed framework will be outlined.

### Wrist kinematics measurement

A simulated sleep experimental protocol (discussed in “Methods” section) was devised to validate the proposed KIDS framework on five healthy human participants. The protocol emulated real sleep by guiding participants through a sequence of 12 common sleep postures, each posture appearing twice but not consecutively, ensuring a randomised distribution of all posture replications. Each participant wore a Bluetooth-enabled miniature wearable sensor module with two IMUs attached, respectively, to the hand and the forearm. The measurements from the sensors were sent over a Bluetooth link to a nearby server on which the KIDS framework was implemented and executed.

For each IMU sensor, its raw measurements were fed into a magneto-inertial sensor fusion algorithm which computed the sensor’s three-dimensional orientation (in the form of a quaternion) referenced to the Earth frame, that is, a coordinate system fixed relative to the directions of gravity and the Earth’s magnetic field. The orientation estimates of the two IMU sensors were subsequently fused to determine the hand-to-forearm relative orientation.

**Figure 2:**
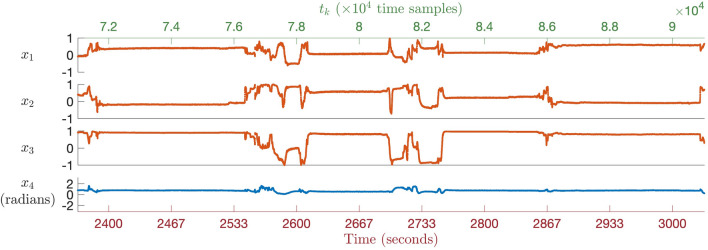
An extract from the hand-to-forearm orientation time series (also referred to as wrist joint kinematic time series), depicted in the four-dimensional axis-angle space, pertains to one of the participants who partook in the simulated sleep protocol. The figure was divided into four subplots: the top three delineate the unitless axis of rotation ($x_1$, $x_2$, and $x_3$), and the bottom subplot corresponds to the angle of rotation ($x_4$) in radians. Recorded at a rate of 30 Hz, each time step corresponds to one-thirtieth of a second. The figure provides a finite time interval for visual clarity, extracted from the complete recording. The simulated sleep protocol involved guiding the participants through a randomised sequence of 12 sleep postures while wearing a sensor module embedded with two inertial measurement units (IMUs) on their left wrist joint. Clear stability was observed in the hand-to-forearm orientation during sustained postures, contrasted with noticeable variations as transitions between different postures occurred.

The relative orientation was subsequently converted from the quaternion space to the more intuitive axis-angle representation for subsequent preprocessing and joint kinematics visualisation. For notation purposes, this paper used ${\varvec{x}} \in \mathbb {R}^4$ $(= x_1 \cdot \hat{i} + x_2 \cdot \hat{j} + x_3 \cdot \hat{k} + x_4 \cdot \hat{w})$ to denote the sensor-measured segment-to-segment orientation in the axis $(\hat{i},\ \hat{j},\ \hat{k})$ - angle $(\hat{w})$ space. The relative orientation time series was indexed using a time stamp vector ${\varvec{t}} = t_1, t_2, \ldots , t_T$. Figure 2 depicts a snapshot of the hand-to-forearm orientation pertaining to one of the participants who partook in the simulated sleep protocol. The orientation exhibited marked stability within periods of sustained sleep postures. Conversely, the transitions between different sleep postures were characterised by conspicuous fluctuations in the orientation.

### Joint kinematics preprocessing and visualisation

The preprocessing of joint kinematics served in obtaining a reduced dimensional representation of hand-to-forearm orientation ${\varvec{x}}$ measured by the wrist-mounted wearable sensor module. The new representation allowed for intuitive three-dimensional visualisation of the joint kinematics, thus making it comprehensible to medical professionals without extensive technical knowledge. Moreover, this reduction in dimensionality implicitly rendered a lower computational cost associated with the subsequent stage of the KIDS framework which employed Bayesian inference, a probabilistic method whose computational complexity rises in direct proportion to the dimensionality of its input data.

**Figure 3:**
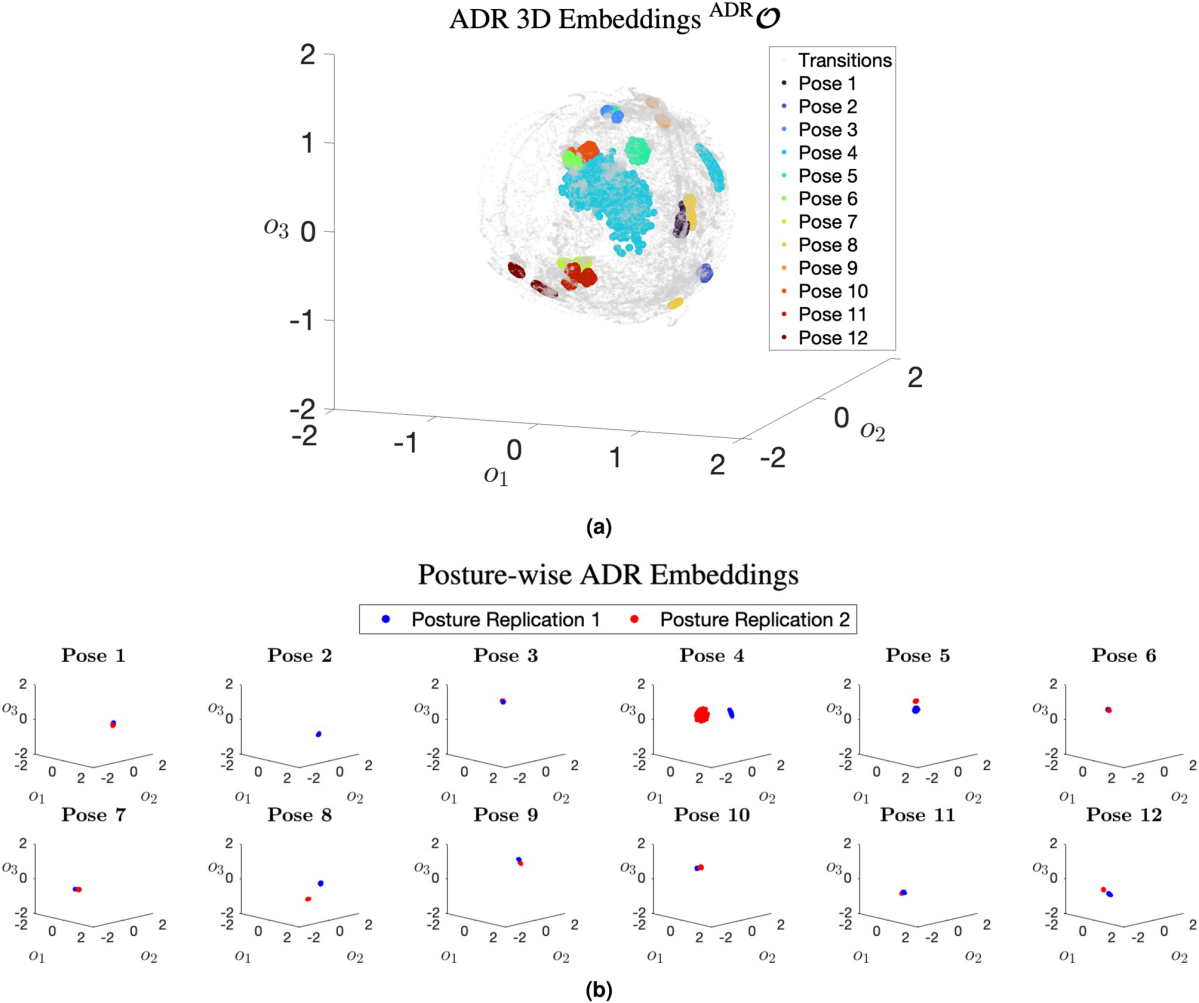
Three-dimensional visualisations of wrist joint kinematic time series, provided by the proposed *analytical dimension reduction* (ADR) method, corresponding to a randomly selected participant. The top subfigure (**a**) illustrates a spherical cloud of embeddings spanning a data acquisition period exceeding 60 min. Latitudinal and longitudinal navigation of the sphere corresponded to distinct axes of rotation, while radial displacement from the sphere’s centre was proportional to the angle of rotation. The embeddings were colour-coded to distinguish between postural transitions and the different sleep postures. These embeddings populate finite regions of the spherical structure, indicating that certain hand-to-forearm orientations are implausible due to the anatomical constraints of the wrist joint. The bottom subfigure (**b**) offers a different view of the three-dimensional embedding space, illustrating only the embeddings corresponding to each of the 12 sleep postures. These embeddings are coloured in red and blue to differentiate between the two replications of each posture performed by the participant as part of the experimental protocol.

As previously explained in “Introduction” section, the dimension reduction process was achieved by implementing one of two methods. The first dimension reduction method was the *Uniform Manifold Approximation and Projection* (UMAP)^31^, an established unsupervised method that facilitated the learning of a lower-dimensional data representation, also referred to as the *embedding space*. While UMAP produced meaningful three-dimensional visualisation of the hand-to-forearm orientations, it had intrinsic limitations and artefacts (see “Methods” section). Consequently, a second dimension reduction method was proposed to analytically approximate the dimensional mapping function of UMAP, with the added benefit of overcoming its limitations and artefacts. Throughout the remainder of this paper, the second method was referred to as *Analytical Dimension Reduction* (ADR). For clarity in notation, the three-dimensional embedding was mathematically denoted by ${\varvec{o}} \in \mathbb {R}^3$ $(= o_1 \cdot \hat{i} + o_2 \cdot \hat{j} + o_3 \cdot \hat{k})$, and the complete embedding dataset $\large \varvec{O}$ encompassed all preprocessed ${\varvec{o}}$ over the timestamp vector, ${\varvec{t}}$.

The ADR was conceptually inspired by UMAP, and was mathematically formulated to map the hand-to-forearm orientations to a thick-crust spherical point cloud. Latitudinal and longitudinal navigation of the point cloud corresponded to distinct axes of rotation, whereas the radial displacement (measured from the cloud’s centre) along the thickness was proportional to the angle of rotation. Figure 3a illustrates over 60 min of hand-to-forearm orientations belonging to a randomly selected participant, as recorded by the miniaturised wearable sensor module. From this visualisation, it can be observed that the three-dimensional embeddings evidently occupy finite regions of the spherical structure, a typical observation since the wrist joint is anatomically constrained, which left some regions unpopulated. To provide a different insight into the point cloud, Fig. 3b portrays only the embeddings corresponding to each of the 12 sleep postures. The posture-wise embeddings demonstrate clear differentiation between the postures, albeit with minor overlaps. Overall, these visualisations supported the hypothesis that single-joint kinematics would be sufficient for the evaluation of postural (in)activity during sleep.

### Sleep posture change detection and temporal segmentation of postural inactivity

The final stage of the KIDS framework jointly addressed two interrelated problems. The first problem was the sleep posture change detection, which focused on identifying the time stamp at which a sustained posture was changed. The current study also referred to this time stamp as “*the changepoint*”. The second problem was the temporal segmentation of postural inactivity, which involved estimating the duration (in time steps) of each sustained sleep posture from its onset up until the next posture changepoint event. In this stage, the framework employed Bayesian inference in conjunction with a changepoint detection logic. On one hand, the Bayesian inference handled the spatial modelling of the three-dimensional embedding time series at each time step to probabilistically infer the durations of postural inactivity periods, based on the assumption that embeddings belonging to a sustained sleep posture have similar statistical attributes (mean and precision). On the other hand, the changepoint detection logic was responsible for the transition from the probabilistic framework to the binary decision-making process that isolated posture changepoints in time.

The metrics for evaluating the performance of the KIDS framework varied according to the requirements of each of the aforementioned two interrelated problems. For the temporal segmentation of postural inactivity task, the *Pearson’s correlation coefficient* (R) was selected to assess the agreement between the predicted and ground-truth durations of periods of postural inactivity. The periods were also referred to as “*inactivity segments*”. The duration of the inactivity segment was the number of time steps between the start and end timestamps of each sustained sleep posture. The start and end timestamps of the ground-truth inactivity segments were manually determined through careful observation of the wearable sensor time series data by an expert human annotator. For the sleep posture change detection task, the $F_{1}$-*score*, *Sensitivity* (Se) and *Positive Predictive Value* (PPV) were the metrics used to evaluate the sleep posture change detection. All the aforementioned metrics were commonly reported in relevant works^28^ and established a good ground for benchmarking.

Table 1 reports the performance metrics for the best-performing variant of the KIDS framework. According to the metrics, the framework demonstrated significant efficacy and reliability across five human participants (P1–P5) in the two interrelated problem domains. For the sleep posture change detection, the framework evidently achieved perfect F$_{1}$-scores (100%) across all participants except for participant (P2), where it dipped slightly to 95%. The three metrics confirmed the superior framework’s ability to detect nearly all instances of sleep posture change.

Regarding the temporal segmentation of postural inactivity, the values of the R metric (in Table 1) indicated a strong positive correlation between the durations of periods of postural inactivity predicted by the KIDS framework and the corresponding ground truth. The correlation coefficient ranged from 0.94 to 0.99, suggesting high temporal segmentation accuracy with a marginal variation across participants.

**Table 1:** The performance metrics resulting from the evaluation of the best-performing variant of the KIDS framework in two interrelated problem domains.

Performance metric	P1	P2	P3	P4	P5	Mean value
Problem domain 1: sleep posture change detection						
F$_{1}$-score	1.00	0.95	1.00	1.00	1.00	0.99
Se	1.00	1.00	1.00	1.00	1.00	1.00
PPV	1.00	0.92	1.00	1.00	1.00	0.98
Problem domain 2: temporal segmentation of postural inactivity						
R	0.94	0.96	0.99	0.97	0.94	0.96

Reported are the metrics across five individual participants (P1 through P5) and their mean values over all participants (rightmost column). In Problem Domain 1, the $F_{1}$-*score*, *Sensitivity* (Se), and *Positive Predictive Value* (PPV) were selected to evaluate the framework’s ability to accurately detect changes in sleep posture. In Problem Domain 2, the *Pearson’s correlation coefficient* (R) was chosen to measure the accuracy of the framework in estimating the durations of periods of postural inactivity.

**Figure 4:**
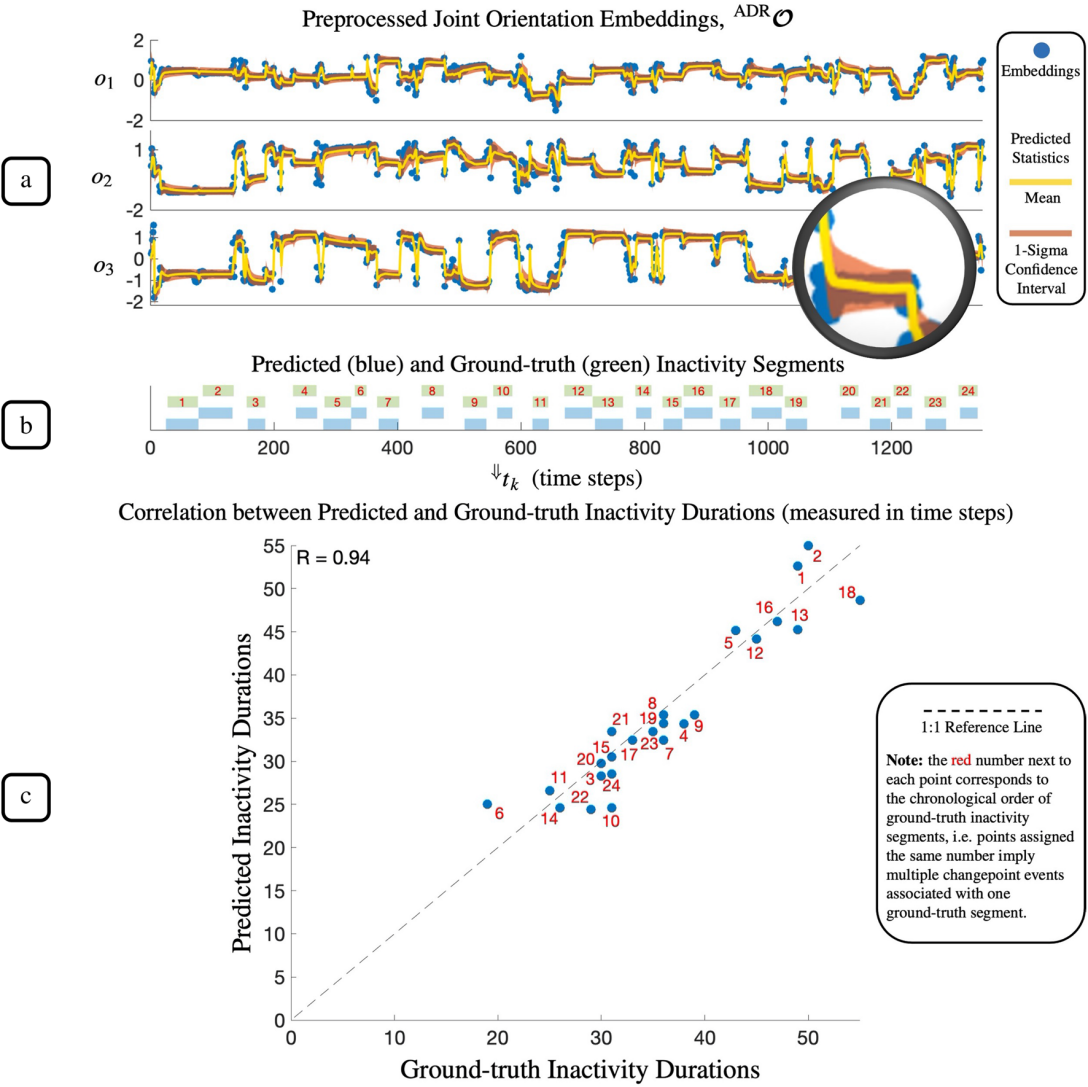
Performance visualisation for the best-performing variant of the KIDS framework applied to the dataset of participant (P1). The top plot (**a**) depicts the downsampled 3D embedding time series (blue points), accompanied by local estimates of the mean (yellow trace) and the 1-Sigma confidence interval (brown strip) as predicted by the Bayesian inference. As shown in the exploded view in (**a**), both statistical attributes gradually converged toward the true distribution of embeddings of each inactivity segment. The middle plot (**b**) presents the framework’s final output, a timeline illustrating the temporal locations of all detected inactivity segments (blue segments) juxtaposed with the ground truth (green segments), demonstrating successful detection of all true inactivity segments without any false positives. The bottom plot (**c**) compares quantitatively the durations of predicted and ground-truth inactivity segments, demonstrating a high degree of agreement (R = 0.94) between both, indicated by proximity to the 1:1 reference line (dashed line).

Figure 4 provides a visual insight into the performance of the best-performing variant of the KIDS framework, as demonstrated on the dataset of participant (P1). Figure 4a shows the downsampled three-dimensional embedding time series, ${\varvec{o}}$, along with localised predictions of the mean and standard deviation, which were by-product statistical attributes of the Bayesian inference. The downsampling of the embedding time series (decimation factor of 100) was performed last at the end of the preprocessing stage for computational advantages explained in the “Methods” section, rendering a new downsampled timestamp vector ${}^{\Downarrow }{}{\varvec{t}} = {}^{\Downarrow }{}{t_1}, {}^{\Downarrow }{}{t_2}, \ldots , {}^{\Downarrow }{}{t_T}$. The predicted statistics confirmed the framework’s ability to model the underlying (hidden) data sampling process unique to each inactivity segment. Upon the onset of each inactivity segment, the 1-Sigma confidence interval (brown strip) gradually converged to the true underlying spread of the segment as shown in the exploded view in Fig. 4a. The observed segment-aware statistical modelling explained the effectiveness of the framework in the detection of sleep posture changepoints and the temporal segmentation of postural inactivity.

The final output of the KIDS framework was a timeline of detected inactivity segments. As an example, this timeline is presented in Fig. 4b which shows the temporal locations of all detected inactivity segments in comparison with the ground truth. As observed from the timeline, all ground-truth inactivity segments were successfully detected with no false positives. Besides the timeline, Fig. 4c quantitatively compares the predicted and ground-truth durations of each detected inactivity segment. It can be observed that all temporal segmentations of inactivity lie in the proximity of the 1:1 reference line, yielding a strong positive correlation coefficient of 0.94.

**Figure 5:**
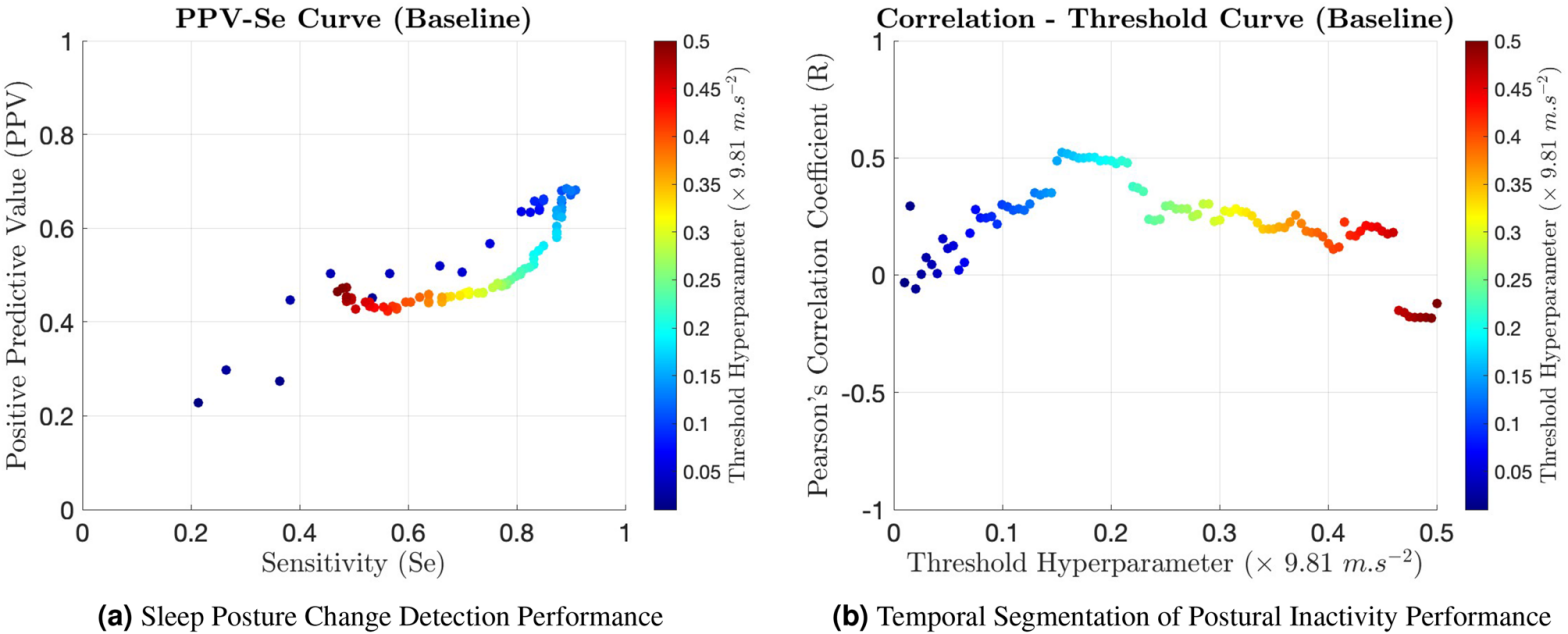
Performance insights into a reported threshold-based baseline approach^17,20,21^ evaluated across two problem domains. The metrics provided were averaged over five participants (P1–P5). Subplot (**a**) delineates the baseline’s sleep posture change detection performance, captured in the dynamic relationship between the Positive Predictive Value (PPV) and Sensitivity (Se) as the classification threshold hyperparameter was adjusted. The PPV-Se pairings were colour-coded based on the hyperparameter’s value. The specified hyperparameter pertained to the maximum relative change in the acceleration magnitude of a hand-mounted sensor, a condition for a sample to be classified as “inactive”. Subplot (**b**) elucidates a similar performance analysis for the temporal segmentation of postural inactivity. The efficacy of the segmentation is indicated by Pearson’s Correlation Coefficient (R) between the predicted and ground-truth durations of inactivity segments in response to a sweeping change in the classification threshold hyperparameter.

### Performance benchmarking of the proposed framework

Following the presentation of the KIDS framework’s results, it is pertinent to benchmark its performance against established methodologies. For this comparison, a threshold-based baseline approach, reported in previous studies^17,20,21^ and aimed at classifying body movement periods, was chosen. To facilitate equitable benchmarking, the baseline approach was slightly adapted to align with the context of the current study. Particularly, the approach was confined to processing data solely from the hand-mounted IMU sensor, in line with previous studies’ singular sensor usage. Additionally, its operational focus was shifted from movement detection to the detection of inactivity segments, thereby enabling a congruous comparison given the ground-truth inactivity segment labels available from the simulated sleep experiment.

Central to this baseline’s decision-making was a threshold parameter, which acted as a condition for variations between successive scalar magnitudes of acceleration. Consequently, the baseline’s performance was assessed by systematically sweeping the value of this parameter from 0 to 0.5 $(\times 9.81\ m\ s^{-2})$, as exhibited in Fig. 5. The performance insights in the figure confirmed that the baseline’s performance was markedly inferior to that of the proposed KIDS framework across both problem domains.

Figure 5a depicts the baseline’s performance in the domain of sleep posture change detection, represented by the PPV-Se curve. This curve amalgamates the PPV-Se pairings, averaged over the cohort of five participants (P1–P5). The PPV and Se metric values revealed a poor-to-mediocre changepoint detection capability, with an F1-score not exceeding 76.60% despite the exhaustive threshold parameter sweep.

On the other hand, Fig. 5b portrays the baseline’s performance in the domain of temporal segmentation of postural inactivity, captured in the R metric averaged across the entire participant set. Across the spectrum of threshold parameter values, the R metric exhibited significant variations. The agreement between the durations of predicted and ground-truth inactivity segments generally suggested the unreliability of the baseline in this domain, ranging from moderate positive correlation (R = 0.52) to negative, poor correlation (R = − 0.18).

In addition to performance effectiveness, computational efficiency was another important criterion for benchmarking. To this end, both the KIDS framework and the baseline were assessed by evaluating their execution time on an approximately 60-min-long dataset from one of the participants, using a 3.8 GHz Quad-Core Intel Core i5 CPU. On average, the baseline approach took 6.10 ms to process the entire dataset, resulting in a per-sample processing time of 0.06 ms. In contrast, the KIDS framework took an average of 106.76 s to process the entire dataset, rendering a per-sample processing time of 98.94 ms as the framework operated on downsampled time series. Despite this difference in processing time, both the framework and the baseline approach achieved real-time performance, as the sample-to-sample intervals with and without downsampling were 300 and 33.33 ms, respectively. This confirmed that the KIDS framework not only attained much higher performance but also did not compromise the real-time computational capability, thereby demonstrating its practicality for further deployments.

## Discussion

### Limitations of previous research

Previous studies predominantly addressed various forms of state detection problems, such as the differentiation between wake and sleep states. Due to the nature of the state detection problem, reported algorithms typically suffered from “*temporal short-sightedness*”; the state detection was often evaluated on a sample or window basis. Nevertheless, few works took the detection information a step further by extracting time-dependent quantities, such as sleep latency and wake after sleep onset^23^. These quantities were estimated rather indirectly from the detection outputs through, for example, accumulating the durations of consecutive windows that were assigned the same state^22^. Consequently, these time-dependent estimates were oftentimes overestimated^28^.

Besides the short temporal span of decision-making in pervasive state detection approaches, methodological limitations and utilitarian constraints also exist. First, threshold-based approaches generally required manual adjustment of hyperparameters to function acceptably across varied participants and sensor devices. Second, classification-based approaches prerequisited sufficiently large labelled datasets for classifier training. Moreover, these sleep datasets were typically imbalanced in nature (disproportionate class-wise sample size) and lacked diversity among participants. Third, while both threshold- and classification-based approaches relied heavily on raw sensor measurements, it was particularly the classification-based approaches that often utilised black-box models. This practice, in turn, contributed to a deficit of explainability which is important prior to adoption in clinical practice.

### Contributions of the KIDS framework

The KIDS framework offered multifaceted contributions that touched upon five primary areas of improvement. First, the *problem space* in which the framework operated and how it compared to research problems typically addressed in reported work. Second, the emphasis on *interpretability* for developing a clear understanding of the framework’s internal workings. Third, the role of *explainability* in communicating the framework’s decisions to both experts and non-experts alike. Fourth, the *temporal span of the framework’s decision-making* and its implications for making robust and contextually informed decisions. Lastly, the *generalisability* of the framework as an indicator of its potential for broader use and adaptation across diverse scenarios and settings. Delineated below are the contributions of the framework in all five areas of improvement.

#### The problem space

Recognising the overlap between sleep posture change detection and temporal segmentation of postural inactivity, the framework was conceived to address the two interrelated problems directly and concurrently. To the best of the authors’ knowledge, this work presents the first simultaneous exploration of both problems, positioning the framework to more comprehensively capture detectable states and their temporal information.

#### Interpretability

Joint movements and orientations are a common language for clinicians. To enhance the comprehensibility of the framework to medical experts and reinforce its relevance to their practice, it was designed to rely purely on processing the measured segment-to-segment orientation about a single body joint, namely the wrist joint, as demonstrated in this manuscript. This joint kinematic profile was utilised to evaluate the whole-body physical activity level.

While it is relatively easy for humans to interpret a joint’s orientation at individual instants of time, it might become less intuitive to visually keep track of the orientation over time. This perceptual complexity stems from the multidimensional nature of the joint kinematic space and the intricacies involved in observing temporal changes. For this reason, not only the input features were crafted to be comprehensible, but also an intermediate stage of the framework was employed, utilising dimensionality reduction to provide a three-dimensional visualisation of the joint kinematic time series. This reduction in dimensionality served dual purposes. On the one hand, it made the complex temporal patterns in the joint kinematic profile more accessible and intuitively graspable by translating them into a visual form that more closely relates to human spatial understanding. On the other hand, the reduction also contributed to lessening the computational burden of the subsequent and final stage of the framework.

#### Explainability

This area emphasises providing justifications for the framework decisions to develop trustworthiness in its decision-making. Simply stated, the decisions of the KIDS framework were the start and end times of sustained postural inactivity, which were evaluated in the tertiary (and last) stage of the framework. In this stage, a fusion between Bayesian inference and changepoint detection logic was utilised, both of which were explainable techniques. Bayesian inference is characterised by its probabilistic nature that accounts for a range of possible outcomes and utilises an evidence-based belief update mechanism, making it one of the established and trustworthy techniques for decision-making. Similarly, the detection logic isolated sleep posture changepoints in time based on a set of explicitly defined rules, which are easy for humans to follow and understand.

#### The temporal span of decision-making

The extended temporal context in which the KIDS framework operated was one crucial aspect that sets the framework apart from previous literature. The framework utilised Bayesian inference, which inherently supports “*sequential learning*”—a process that integrates previous knowledge and continually updates beliefs as new information becomes available over time. Harnessing this capability for sequential learning, the framework conducted a *probabilistic spatial modelling of time series* which captured how the statistical attributes of measured joint kinematics evolved over time. By incrementally constructing this statistics timeline, the framework was subsequently able to infer both the specific moments of change (changepoints) and the durations of postural inactivity periods (durations of sustained statistics) within the joint kinematic time series. The working principle of the framework was in stark contrast to earlier approaches, which operated on individual samples or short time windows and had a restricted ability to recognise the broader patterns within time series data.

#### Generalisability

There were two primary hurdles associated with the development and implementation of earlier approaches in the literature: (1) the prerequisite of large, balanced and labelled training data and (2) the meticulous tweaking of hyperparameters for performance stability. A substantial advantage of the proposed framework was that both hurdles did not apply to it. In regard to the first hurdle, the development of the framework did not involve the collection of training data from human participants. For the second hurdle, all framework hyperparameters were fixed across all participants and no finetuning was conducted during the development stage. This hyperparameter relief was the outcome of utilising hyperparameters that were independent of human behaviours, such as the movement intensity often used in threshold-based approaches). Alternatively, the framework benefited from two strategies. The first strategy ensured that the definition of hyperparameters stemmed purely from a methodological design perspective. For example, the design of the intermediate dimension reduction-based stage of the framework benefited from the targetted spherical topology in the three-dimensional embedding space. The second strategy was utilising the adaptive Bayesian inference approach, which enabled the framework to learn effectively from sensor measurements as they become available over time despite the presence of hidden variables, such as subject-to-subject variability and sensor noise.

### Assumptions of the KIDS framework and their validity

The KIDS framework laid down two primary assumptions related to the monitoring of joint kinematics and its temporal resolution. The following text explains each assumption and the rationale behind it.

**Assumption 1**: *the kinematics of a single joint reflect the whole-body activity level*. The tracking of multiple body joints during sleep could be uncomfortable and technically unjustifiable, depending on the purpose of the study. For the detection of sleep posture changepoints and the temporal segmentation of postural inactivity, it was logical to investigate whether monitoring the kinematics of a single joint would be sufficient to jointly address the two problems. The hand is probably one of the most moved parts of the human body, and being lightweight, it potentially carries much of the information on body mobility during sleep. Therefore, as a starting point, this paper exploited the feasibility of utilising the kinematics of the left wrist joint alone.

**Assumption 2**: *the measured joint kinematics need not have a high temporal resolution*. While sensors with high refresh rates are capable of capturing higher-order kinematics, it can be argued that sleep, being a dormant state of the human body, does not always require an ultra-fast algorithm to detect changes in physical sleep behaviour. Moreover, excessive computations would compromise power efficiency and real-time performance, which are desirable criteria for portable sleep monitoring devices. Assuming that every posture transition would incur a permanent change in the estimated hand-to-forearm orientation, the requirement for a frequently updating framework was redundant. Consequently, the KIDS framework safely incorporated the downsampling of the three-dimensional embeddings. As a result, the tight time constraint on the computation cycle of the framework was substantially relaxed, allowing for the adoption of more advanced approaches, such as Bayesian inference, without compromising real-time performance.

The performance evaluation for the best-performing variant of the KIDS framework confirmed that the two aforementioned assumptions held true, subject to a minor contingency (covered below). According to Table 1, the framework achieved high performance metrics across all five participants in the two interrelated problem domains. Specifically, the framework detected nearly all sleep posture changepoints (mean F$_{1}$-score = 99%) and demonstrated supreme efficacy in estimating the durations of periods of postural inactivity (mean R = 0.96%).

### Limitations of the KIDS framework and avenues for future research

While the results from the experimental protocol suggested the promising potential of the framework, it is important to recognise its limitations and, where possible, potential solutions.

The KIDS framework in its current form utilised a bespoke wearable sensor module^11^ to measure the segment-to-segment orientation about a single body joint. In this work, this quantity was considered an indicative measure of the whole-body physical activity level during sleep. However, it is worth noting that most commercial devices, such as smartwatches, typically feature a single IMU sensor and are affixed to only one body segment. Nevertheless, in instances where dual-IMU sensing is not supported by the device, the KIDS framework can still be adapted to utilise a single segment’s orientation instead of the segment-to-segment relative orientation. This alternative orientation can be obtained in a similar manner by employing the Madgwick filter to fuse the IMU measurements available in the device.

Based on a detailed analysis of the KIDS framework, it is important to highlight a potential source of failure that may affect the framework’s ability to detect sleep posture changepoint events. Since the permanent change in the joint kinematics was crucial for the framework to identify changepoint events, the detection turned out to be more challenging in rare cases where the wrist joint orientation does not change significantly across two consecutive sleep postures, particularly if the intermediate posture transition was ephemeral. Though the best-performing variant of the framework detected nearly all changepoint events across all participants, the aforementioned failure case was close to happening once during a posture transition. In this particular case, the framework demonstrated a slightly “weaker” detection event, but the change in posture was detected nevertheless.

While the sequential learning of Bayesian inference lent the framework adaptability to hidden variables, such as subject-to-subject variability, it might incur additional computational cost for time series that are too long. This limitation was not encountered in this study as only a simulated sleep protocol was implemented, which was shorter than real sleep by orders of magnitude. In practice, there are workaround solutions to this limitation which include, for example, pruning the time series and restarting the framework once the duration of the past time series exceeds a predetermined value, say an hour.

While this study elucidated several key dimensions of the proposed framework, it simultaneously unveiled partially explored territories for further scientific inquiry. Herein presented two avenues for improving the framework in the future. First, the framework could be applied to kinematics observed from various body joints, such as the ankle, in order to derive formal recommendations for ideal sensor placement. Second, the validation of the framework could be extended to real sleep scenarios with larger cohorts of participants, including individuals with sleep disorders.

## Methods

To foster clarity and systematic comprehension of the results reported in this paper, this section provides an in-depth overview of the KIDS framework at first, spotlighting its purpose, design rationale, and deployment procedures. This preliminary discussion provides the conceptual groundwork for the second, more detailed subsection on the methods pertinent to the study and the proposed framework. The third subsection presents aggregative results from the variants of the framework, in addition to performance benchmarking analysis.

### The KIDS framework: design rationale and operational overview

The proposed KIDS framework, conceived as a plug-and-play wearable system, was specifically developed to facilitate unobtrusive and automated analysis of postural (in)activity during sleep. Through gauging and analysing clinically meaningful kinematics of a single body joint, the framework presented a comprehensive solution to two interconnected problems simultaneously: sleep posture change detection and the temporal segmentation of postural inactivity. The output of the framework primarily comprised a detailed timeline of segmented periods of postural inactivity (refer to Fig. 4b for an illustrative example), clearly indicating the start and end timestamps of each inactivity segment. In addition to the timeline, the framework provided a complementary three-dimensional visualisation (exemplified in Fig. 3a), elucidating the temporal variations in the joint kinematics and enhancing comprehensibility to both clinicians and lay users. The framework could be particularly useful for clinicians probing sleep-associated behaviours and disorders. There are several use cases of the framework in sleep medicine, ranging from basic sleep quality assessments to more complex investigations into the implications of physical sleep behaviour for the diagnosis of underlying health conditions, such as musculoskeletal disorders.

In a real sleep scenario, it was envisaged that the deployment of the KIDS framework would follow a systematic and user-friendly procedure. Initially, the subject would attach the miniature wearable sensor module, provided by clinicians beforehand, to their wrist joint, specifically by affixing the module’s two IMUs onto their hand and forearm segments, respectively. The IMUs would only need to be approximately aligned to their respective segments. Subsequently, the subject would power on both the sensor module and a proximate server, such as a smartphone, and then a Bluetooth connection would be established between the two devices accordingly. This connection would serve as the medium for the real-time transmission of sensor measurements. Owing to the framework’s design, which eliminated the need for preliminary training data collection, the subject would seamlessly proceed with their usual sleeping routine. Throughout the night, the collected measurements would be continuously transmitted to the server. Upon awakening, the subject would terminate the Bluetooth connection and deactivate the sensor module. The amassed data would then be dispatched to a cloud server where subsequent data processing, analysis and visualisation would take place. Finally, the analytical report delineating the subject’s overnight postural (in)activity would be readily accessible via an online platform to both the subject and their reporting clinician, promoting transparency in care and facilitating timely and informed decisions.

The research outlined herein pivoted on a simulated sleep protocol, devised to assess the performance of the KIDS framework in conditions approximating real sleep settings. The ensuing subsection details the stages of the proposed methodology, spanning from the initial data acquisition to the culmination of the framework operation.

### Comprehensive walkthrough of the methodology

This section provides a detailed account of the comprehensive methodology utilised in the research presented. The discussion is organised in alignment with the workflow, commencing with the participant study, which encompassed the design and execution of the simulated sleep protocol. Subsequently, it delves into each of the three stages of the KIDS framework, elucidating the methods employed in each stage. The methodology encompasses all the variants of the proposed framework, including the best-performing variant. Moreover, the discussion explains the rationale behind the design choices and provides comparisons and reflections, where applicable.

#### Participant study: simulated sleep protocol

The study involved a simulated sleep protocol which was devised to emulate realistic sleep settings, thereby ensuring the applicability of the subsequent analysis of the proposed framework performance.

Five healthy adult participants (age: 36 ± 15.8 years, height: 169 ± 11 cm, body weight: 72.8 ± 23.2 kg) willingly partook in the study upon providing informed consent. The methods were carried out in accordance with relevant guidelines and regulations and all experimental protocols were approved by The University of Liverpool Research Ethics Committee (review reference: 9850). Each participant attached a bespoke wearable sensor module to their left wrist, with its two integrated IMUs affixed to the hand and forearm segments, respectively. The sensor module was designed to gauge the hand-to-forearm relative orientation, also referred to as wrist joint kinematics, and to transmit these data to a proximate server at a rate of 30 Hz. Once the sensor module was set up and running, the study proceeded with the simulated sleep protocol, in which the participant was guided through a randomised sequence of 12 common sleep postures. To further assist the participant through the posture replication procedure, a leaflet containing pictures of the 12 sleep postures was initially handed to them (definitions of postures can be found in previous work^11^). Each posture appeared twice in the sequence, but not consecutively. This random posture shuffling strategy was adopted to ensure statistical independence among samples across the dataset and to resemble a realistic sleep scenario. Throughout the study, the duration of sustained postures and intermediate transitions varied depending on the comfort of the participants and their need for guidance from the on-site researcher.

For the collected data to be fit for the purpose of evaluating the performance of the KIDS framework, it was imperative to acquire high-quality ground-truth labels. The choice of the ground truth definitions was primarily determined by the problems framework addressed: the sleep posture change detection and the temporal segmentation of postural inactivity. To this end, the start and end timestamps for each posture formed the minimum set of ground-truth labels required to assess the performance of the framework comprehensively. The acquisition of these labels was conducted over two steps. First, the researcher on site recorded the approximate boundary timestamps for each posture during the simulated sleep protocol. Second, these approximate timestamps were refined by an expert annotator by manually examining the collected wearable sensor time series data, pinpointing the exact start and end timestamps for each posture.

With this joint kinematics dataset in place, the following section transitions to a technical account on the measurement of wrist kinematics; the foundational pillar of the proposed framework.

#### Wrist kinematics measurement

The measurement of meaningful joint kinematics was a distinctive highlight that set the presented study apart from previous work reported in the literature. This section elaborates on the instrumentation and data processing methods that enabled the measurement of these kinematics.

##### Bespoke wearable sensor module

 The participant study employed a custom-made wearable sensor module, specifically designed to measure the left wrist joint kinematics, or alternatively the hand-to-forearm relative orientation. In a previous work^11^, four such modules were affixed to extremity joints (wrists and ankles) to provide a comprehensive sleep posture classification. However, for a start, this study explored the feasibility of whether the kinematics of a single joint would be sufficient to evaluate postural (in)activity during sleep. Consequently, the wrist joint was selected for the analysis due to the rich information it potentially carries on body mobility, being one of the most moved and lightweight parts of the human body.

The custom-made sensor module offered dual-segment orientation tracking across the wrist joint, enabled by two embedded IMU sensors mounted on the hand and forearm respectively. The IMU model was the BNO055 (Bosch Sensortec GmbH, Reutlingen, DE). Both IMU sensors were managed by a single ESP32-WROOM-32D microcontroller (Espressif Systems Shanghai Co Ltd, Shanghai, CN) featuring Bluetooth connectivity for wireless data transmission. At about 6 cubic centimetres in volume for each IMU case, the sensor module was sufficiently slim and small for wearability during sleep. Prior to each in-vivo experiment, all IMU sensors were calibrated according to standard procedures^32,33^ to estimate and reduce errors owing to constant bias, scale factors, cross-axis sensitivity and response nonlinearity.

##### Magneto-inertial sensor fusion

 A dual-stage fusion approach, encompassing both *intra- and inter-sensor fusion*, was adopted in the work. The orientation of each IMU sensor was estimated using a magneto-inertial sensor fusion algorithm, termed as intra-sensor fusion. Since each IMU sensor was securely attached to its respective body segment, each IMU sensor’s estimated orientation was a function of the orientation of the segment to which it was affixed. Utilising orientations derived from the two IMU sensors, an inter-sensor fusion was subsequently employed to determine the relative orientation of the hand to the forearm across the wrist joint, i.e. the hand-to-forearm orientation.

For intra-sensor fusion, the Madgwick filter^34^, a computationally efficient and robust sensor fusion algorithm, was used to fuse the magneto-inertial measurements from the IMU sensor, providing a filtered estimate of the sensor’s absolute orientation with respect to the Earth reference frame in the quaternion space. The utilisation of the quaternion space contributed to removing singularities from the orientation space. In regard to inter-sensor fusion, kinematic transformations facilitated the derivation of hand-to-forearm relative orientation by referencing the hand (child segment) IMU orientation to that of the forearm (parent segment). Comprehensive mathematical underpinnings of this fusion process are available in an earlier work^11^. Following this, the relative orientation was transmuted from the quaternion space to the axis-angle space, where a unique ${\varvec{x}}(t_k) \in \mathbb {R}^4$ existed at each discrete time step $t_k$. Such an axis-angle rendition of joint kinematics was more interpretable than its quaternion counterpart and facilitates the derivation of meaningful insights, as demonstrated in previous work^11^.

#### Joint kinematics preprocessing and visualisation

While the four-dimensional axis-angle representation provided a detailed snapshot of the wrist kinematics at specific instances, it did not aptly convey the evolution of these kinematics over time. Dimension reduction, with its successful applications spanning diverse areas from wearable sensing^11^ and speech processing^35^, to knowledge exchange^36^, offers a compelling solution to this challenge. Consequently, the second stage of the KIDS framework employed preprocessing methods to reduce the dimensionality of the kinematic time series, thereby providing visualisations that promote the intuitive comprehension of the measured kinematics to domain specialists and lay users alike. Additionally, the reduced dimensionality contributed to computational gains which would be evident at the ultimate stage of the framework.

Presented in this section are two distinct dimension reduction methods based on unsupervised learning and analytical transformation, respectively. Following this, the section ends with the framework’s last preprocessing step which involved the downsampling of the dimensionally reduced kinematic time series.

##### Unsupervised learning-based dimension reduction (option 1 for dimension reduction)

 Unsupervised learning-based dimension reduction captures the essence of high-dimensional data without the need for labels, making this category of methods apt for simplifying complex datasets. Within this category, the *Uniform Manifold Approximation and Projection* (UMAP)^31^ stands out as a prominent nonlinear dimension reduction method. It gradually constructs a lower-dimensional force-directed graph that captures the patterns of the original high-dimensional dataset. UMAP’s advantage lies in its ability to deal with nonlinear data manifolds while preserving both local and global data structures within the low-dimensional embedding space. It seamlessly accomplishes this through two core stages: first, it identifies nearest neighbours to form a neighbour graph, and second, it learns the dimensionally reduced representation through iterations of minimising a dedicated cost function. A thorough discussion on the underpinnings of UMAP is available in its original paper^31^.

The use of UMAP in the current study was distinct from the common use of dimension reduction within the literature of human motion analysis^37–40^. Previous works utilised dimension reduction methods to transmute pre-collected data into visualisable low-dimensional embeddings. While these visualisations were useful in discovering data patterns, they were mainly applicable to the processed dataset and they might fail to generalise to unseen data if the new samples were drastically different from the pre-collected ones. Consequently, the implementation of UMAP in this work was performed over two steps to promote its generalisability to as many, if not all possible, orientations in the axis-angle space. The first step involved presenting a synthetic orientation dataset to UMAP for it to learn the mapping from the four-dimensional axis-angle space to a three-dimensional embedding space. The synthetic orientation dataset was carefully constructed such that it uniformly sampled the axis-angle orientation space (given a predefined resolution), thereby enabling UMAP to construct a reliable neighbour graph. Subsequently, the second step utilised the pre-learnt dimensional transformation of UMAP to import the sensor-measured wrist kinematics into the same embedding space.

**Figure 6:**
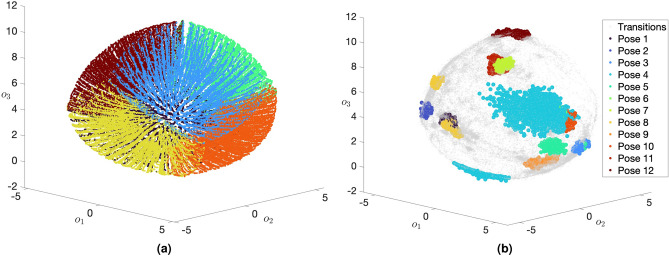
Visualisations of both a synthetic orientation dataset (left) and the sensor-measured wrist kinematic time series (right) in a three-dimensional embedding space, as provided by the *Uniform Manifold Approximation and Projection* (UMAP) method. Initially, synthetic orientations uniformly sampled from the axis-angle space were presented to UMAP to enhance its generalisability to (unseen) sensor-measured orientations in subsequent uses. Subplot (**a**) presents the synthetic orientation embeddings, colour-coded according to the directions of their respective axes of rotation ($\pm x_1$, $\pm x_2$, and $\pm x_3$). Notably, the topology of these embeddings resembled a thick-crust, egg-like structure, where longitudinal and latitudinal navigations of this structure were associated with varying axes of rotation, while radial displacements corresponded to varying angles of rotation. Following the initial use of synthetic data, UMAP was reused to import the wrist kinematic time series, obtained from a randomly selected participant, into the same pre-learnt embedding space as depicted in subplot (**b**). These embeddings were colour-coded to distinguish between postural transitions and the different sustained sleep postures during the simulated sleep protocol. Only finite regions of the egg-like structure were populated with embeddings, a result of the anatomical constraints inherent to the wrist joint.

Illustrations of UMAP’s three-dimensional visualisations, both for the synthetic orientation dataset and for a participant’s sensor-measured wrist kinematics, are depicted in Fig. 6a,b respectively. The three-dimensional embeddings formed a thick-crust structure that resembled the shape of an egg. In Fig. 6a, the embeddings were coloured based on the direction of their respective axes of rotation, namely $\pm x_1$, $\pm x_2$ and $\pm x_3$. Based on this colour convention, it was evident that UMAP associated longitudinal and latitudinal navigations of the egg-like structure with different axes of rotation. Additionally, different angles of rotation corresponded to varying radial displacements, as measured from the centre of the structure. When importing over 60 min of sensor-measured wrist kinematics pertinent to a randomly selected participant into this embedding space, Fig. 6b shows that only finite regions of the space were populated due to the anatomical constraints discussed in “Results” section. Moreover, the populated regions revealed a decent separation between the 12 sleep postures replicated throughout the simulated sleep protocol.

The uniform sampling of the axis-angle space was a key concept in the creation of the synthetic orientation dataset. Since the axis-angle representation comprises two components, the axis and the angle of rotation, the domain of each component was sampled uniformly in isolation, and then all possible axis-angle combinations were generated to form the complete synthetic orientation dataset.

Sampling the axes of rotation could be simply performed by sampling the surface of a three-dimensional sphere. However, the naiv̈e approach of choosing equidistant latitudinal and longitudinal angles would render denser axes of rotation near the poles and sparser ones near the equator. Consequently, a computer graphics pipeline was employed to generate a set of uniformly sampled axes of rotation. The pipeline commenced with the construction of a cubic structure, whose vertices were then meticulously projected onto a unit sphere containing the cube. In the first step, a *procedural mesh generation* technique was used to construct the unit cube with face vertices, ${\varvec{v}}_f = \{x_f, y_f, z_f\}$. Following this, the ellipsoidal projection

1 $$\begin{aligned} {\varvec{v}}_e = \begin{bmatrix} x_e&y_e&z_e \end{bmatrix} = \begin{bmatrix} \ x_f\sqrt{1 - \frac{1}{2}\ y_f^2 - \frac{1}{2}\ z_f^2 + \frac{1}{3}\ y_f^2z_f^2}&y_f\sqrt{1 - \frac{1}{2}\ x_f^2 - \frac{1}{2}\ z_f^2 + \frac{1}{3}\ x_f^2z_f^2}&z_f\sqrt{1 - \frac{1}{2}\ x_f^2 - \frac{1}{2}\ y_f^2 + \frac{1}{3}\ x_f^2y_f^2}\ \end{bmatrix} \end{aligned}$$

was utilised to project all ${\varvec{v}}_f$ onto the surface of a unit sphere, to ensure that the projections were evenly distributed over the surface of the sphere. The projected vertices, ${\varvec{v}}_e$, represented the synthetic axes of rotation, and were then exported into a *comma-separated values* file. All procedural three-dimensional modelling was implemented in the C# programming language and visually produced in Unity$^{\copyright }$ (Unity Technologies Inc., California, US).

Moving to the synthesis of the angles of rotation, an equidistant set of 36 angles, $\{\frac{36}{36}\pi ,\frac{35}{36}\pi ,\frac{34}{36}\pi ,\ldots ,\frac{1}{36}\pi \}$, were considered as eligible values. Finally, both the axes and angles generated previously were fused to form the synthetic orientation dataset. To this end, the exported file containing all ${\varvec{v}}_e$ was subsequently imported into MATLAB$^{\copyright }$ (The MathWorks, Massachusetts, US). Therein, an orientation dataset generator script exhausted all unique combinations between the axes and angles of rotation, ultimately yielding a total of 48,600 orientations. Further details on the computer graphics pipeline are available online in Supplementary Methods titled “Computer graphics pipeline”.

While the unsupervised learning capability of UMAP produced meaningful visualisations of kinematics, the embedding space turned out to be weakly constrained which was evident in the geometrical artefacts inherent to the egg-like structure. According to Fig. 6, the egg-like structure had different stretch factors along the three pseudo-dimensions, $o_1$, $o_2$ and $o_3$, in addition to having a non-zero offset from the origin of the embedding space. The implication of the unequal stretch along the three dimensions was that the difference between embeddings at compressed regions of the structure could be significantly larger than that between embeddings at expanded regions, even if the two cases were equivalent in the original axis-angle space. Therefore, the following section presents a second dimension reduction method that analytically approximated UMAP’s dimensional transformation, whilst maintaining full control over the embedding space to resolve the geometrical artefacts discussed above.

##### Analytical dimension reduction (option 2 for dimension reduction)

 Building upon the observations from the UMAP’s visualisations, this section proposes an enhanced alternative to UMAP, the *Analytical Dimension Reduction* (ADR). Distinctively, ADR was not a standalone dimension reduction method; instead, it was primarily inspired by UMAP’s visualisations and exclusively targeted one task which was reducing the four-dimensional axis-angle orientations to a more tractable three-dimensional space. Unlike UMAP, which relied on unsupervised learning, ADR harnessed pure mathematics and geometry to achieve the sought dimensional transformation. The objective behind ADR was to generate a perfectly structured thick-crust sphere in the embedding space, devoid of any geometrical artefacts, thereby ensuring a more consistent and standardised representation in comparison to UMAP. For visualisation purposes only, the synthetic orientation dataset, which was previously used to facilitate UMAP learning the dimensional transformation, was re-visualised by ADR, as shown in the perfectly sampled spherical structure in Fig. 1. Depicted in Fig. 3a are the embeddings corresponding to over 60 minutes of wrist kinematic time series for a randomly selected participant.

Motivated by the topology of the UMAP’s embeddings, the ADR sphere was configured with inner and outer radii, forming a thick-crust sphere. This spherical configuration not only retained the meaningfulness of visualisation offered by UMAP but also rectified its inherent geometrical artefacts. Within this spherical structure, traversing in the latitudinal and longitudinal directions connoted distinctive axes of rotation, whereas the radial displacement along the crust’s thickness was proportionate to the change in the angle of rotation.

The procedure of dimensionally transforming an axis-angle orientation was performed over two steps. The first step determined the radial displacement from the centre of the sphere, followed by the second step which defined all three cartesian coordinates of the embedding at that radial displacement.

The radial constraints of the ADR sphere were set between an inner radius, ${}^{i}{}{r_s} = 1$ unit length, and an outer radius, ${}^{o}{}{r_s} = 2$ unit lengths. The displacement between these two endpoints reflected a change in the angle of rotation from 0 to $\pi$ radians, respectively. With this geometric correlation, the embedding’s radial displacement, ${}^{i}{}{r_s}$, could be interpolated based on the sensor-measured angle of rotation:

2 $$\begin{aligned} r_s = \frac{1}{\pi } x_4 + {}^{i}{}{r_s} \end{aligned}$$

The latitudinal and longitudinal navigation across the spherical structure corresponded to differently oriented axes of rotation as defined by the components $x_1$, $x_2$ and $x_3$. Building on this definition and taking into account the radial displacement from Eq. (2), an arbitrary ADR embedding, ${}^{\text {ADR}}{}{\varvec{o}}$, can be described by

3 $$\begin{aligned} {}^{\text {ADR}}{}{\varvec{o}} = \begin{bmatrix} o_1&o_2&o_3 \end{bmatrix} = r_s \begin{bmatrix} x_1&x_2&x_3 \end{bmatrix} \end{aligned}$$

The implementation of ADR hinged on mathematical formulations to achieve the geometric configuration aforementioned. Crucially, due to these mathematical confines, ADR ensured no embeddings ventured beyond the defined thick-crust spherical bounds.

##### Downsampling of dimensionally reduced wrist kinematics

An inherent inverse correlation would be expected between the length of the three-dimensional embedding time series (in time steps) and the computational efficiency of the methodology utilised in the ensuing (and ultimate) phase of the KIDS framework. Put differently, the larger the number of time steps, the tighter the time constraints would be for the employed algorithm. Under such circumstances, the algorithm’s capacity would have to be compromised, and only rudimentary to moderately complex algorithms would be rendered viable, akin to the pervasive naiv̈e threshold-based approaches.

A fact is that sleep patterns are characterised by extended periods of inactivity. Hence, downsampling represents a good workaround solution to curtail computational demands and data storage requirements for wearable devices. Capitalising on this insight, the temporal resolution of the complete embedding dataset, $\large \varvec{O}$, was reduced by a decimation factor of 100. This step produced a new downsampled timestamp vector ${}^{\Downarrow }{}{\varvec{t}} = {}^{\Downarrow }{}{t_1}, {}^{\Downarrow }{}{t_2}, \ldots , {}^{\Downarrow }{}{t_T}$. Notably, this downsampling step was implemented irrespective of the selected dimension reduction method.

#### Sleep posture change detection and temporal segmentation of postural inactivity

The previous stages of the framework shed light on the acquisition and treatment of wrist kinematics. Recalling the current study’s main purpose which was to leverage these kinematics in facilitating an automated analysis of postural (in)activity during sleep, this section presents the ultimate stage of the proposed framework, incorporating its decision-making core wherein these measurements culminated in purposeful outcomes. The methods herein are discussed in a top-down manner. Firstly, the section provides a clear formulation of the problems addressed by the framework, accompanied by a succinct overview of the strategy for tackling them. Following this foundation, the discussion proceeds with elucidating the internal workings of each step within this strategy.

##### Problem statement and strategic plan

 The ensuing discussion would not only clarify the nature of the problems addressed by the KIDS framework but also provide a structured overview of the methodological components designed to solve them, thereby establishing a clear foundation for the methods covered subsequently.

The KIDS framework was conceived to tackle two closely related problems. Firstly, there was the sleep posture change detection problem, which entailed pinpointing the posture changepoints, or the moments when a participant shifted from one prolonged sleep posture to another. Concurrently, the second problem revolved around the temporal segmentation of postural inactivity, which aimed to estimate the duration, counted in time steps, of each inactivity segment. More explicitly said, this duration represented the time interval starting from the onset of a sleep posture until the subsequent posture changepoint. Consequently, a joint solution to these problems would manifest as a clear timeline, demarcating the beginning and end of each period of postural inactivity, effectively denoting the boundaries of sustained sleep postures.

With the three-dimensional visualisations availed by UMAP and ADR, it was observed that changes in sleep postures were associated with considerable changes in the spatial arrangement of the embeddings. Capitalising on this observation, the third stage of the framework concentrated on modelling the evolving spatial distribution of these embeddings. The intent was to proficiently predict when posture changepoints occurred and to segment periods of postural inactivity accordingly.

To achieve this, the strategy employed four (three essential and one recommended) methodological components—Bayesian inference, probabilistic point estimation, an enhancement step, and changepoint detection logic. These were instrumental in transitioning from a probabilistic decision-making space with multiple potential outcomes to a single binary outcome.

The Bayesian inference was utilised to recursively derive a posterior distribution over the duration of postural inactivity based on the observed three-dimensional embeddings up to each point in time. Subsequently, through probabilistic point estimation, the most probable value or point estimate for the duration of postural inactivity was determined for each time step, given its posterior distribution. In scenarios where sleep postures were sustained, this point estimate would grow linearly in time. However, a change in the sleep posture would trigger this estimate to reset nearly to zero. This resetting pattern was instrumental in facilitating the detection of changepoints subsequently. An auxiliary enhancement step was incorporated to refine the timeline of point estimates. The aim here was to reinforce the magnitude of change noticeable during transitions from one posture to another. The significance of this enhancement to the overarching performance of the KIDS framework will be highlighted through an ablation study in the concluding part of “Methods” section. Lastly, a changepoint detection logic was devised to isolate posture changepoints in time through discerning instances when the point estimate was reset. Each changepoint marked the end of an inactivity segment whose duration was equal to the value of the point estimate preceding the changepoint.

By coherently weaving together these methodologies, the KIDS framework was poised to effectively estimate the temporal bounds of postural inactivity. With the problems and strategic plan now clearly outlined, the following discussion will elucidate the intricacies of each of the four methodological steps.

**Figure 7:**
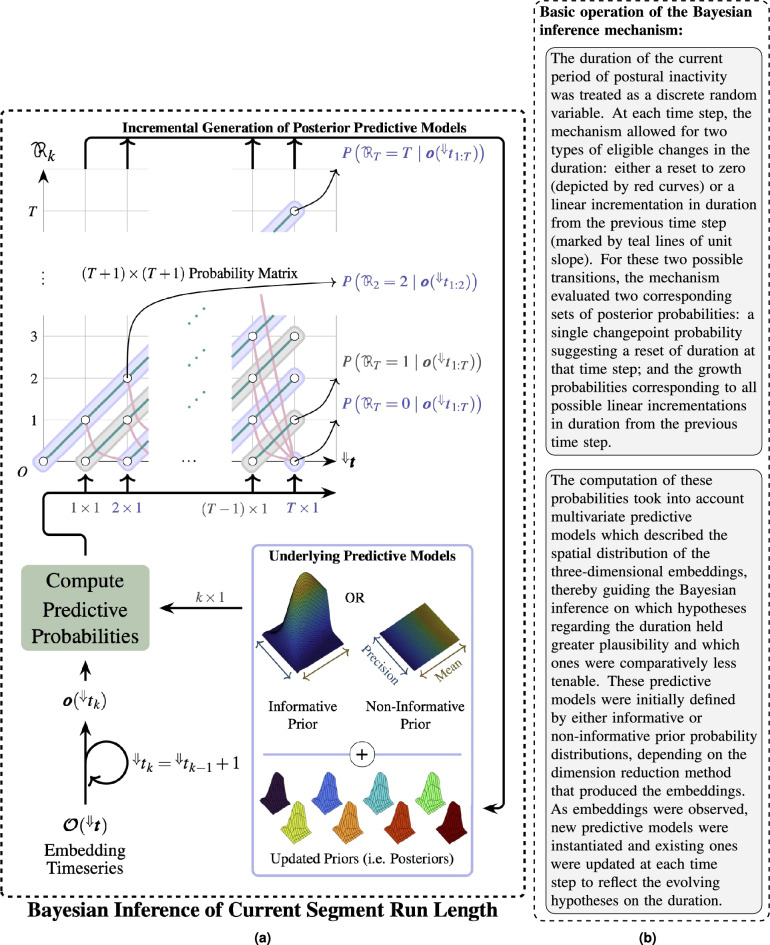
Graphical illustration (**a**) coupled with explanatory text (**b**) about the Bayesian inference mechanism used to infer the posterior probability distribution over the duration of the current period of postural inactivity at each time step.

##### Bayesian inference (Step 1)

 Bayesian inference offered a means to probabilistically determine the duration of postural inactivity based on the observed embeddings up to each point in time.

The Bayesian inference method employed in this work was largely adapted from a previous study^41^, which reported a probabilistic mechanism for evaluating all possible hypotheses on the length of homogeneous data segments, constructed from discrete observations sharing similar statistical attributes. In the present work, this method was employed to infer the number of consecutive time steps over which the three-dimensional embeddings manifested similar statistical attributes. This inferred quantity was referred to as the *current segment run length*, , highlighting the emphasis on evaluating only the length of the current segment of postural inactivity at each time step ${}^{\Downarrow }{}{t_k}$.

While this method determined the length of the current segment, the inference of  nonetheless considered all past observations in the probabilistic decision-making, including those observations from previous segments. This was a primary advantage over the previously reported threshold- and classification-based approaches, which were characterised by a generally limited temporal span for decision-making due to their operation on a sample or window basis.

While a comprehensive elucidation of this Bayesian method can be found online in Supplementary Methods titled “Bayesian inference of current segment run length”, a succinct overview of its constituent steps is nonetheless provided here to provide a high-level understanding of its underlying operational mechanics depicted in Fig. 7.

At the initial time step, ${}^{\Downarrow }{}{t_0}$, the method was presented with two primary inputs: the three-dimensional embedding time series, and the predictive probability distribution describing their spatial distribution. The distribution was modelled as a multivariate Gaussian process, with a mean vector and a precision matrix as model hyperparameters. By the end, at ${}^{\Downarrow }{}{t_T}$, the method yielded two outputs. Firstly, the posterior probability distribution over  given all observed embeddings. This posterior described the probabilities for all $T+1$ hypotheses on , from  to . The other output was a set of $T+1$ predictive distributions corresponding to all possible hypotheses on the number of past embeddings that could contribute to modelling the spatial distribution of the embeddings at ${}^{\Downarrow }{}{t_T}$. These models vary from the completely agnostic model (given ) to the complete-history-aware model (given ).

Moving on to the mechanics of the Bayesian inference, there were notably two types of eligible changes in the current segment run length at each time step; either a reset to zero or a linear incrementation from the length at the previous time step. To this end, the Bayesian inference evaluated two types of posterior probabilities on  at each time step, ${}^{\Downarrow }{}{t_k}$. The first type was the changepoint probability, , which corresponded to a single hypothesis suggesting a changepoint occurring at ${}^{\Downarrow }{}{t_k}$. The second type comprised the growth probabilities, , which corresponded to all hypotheses suggesting linear incrementations from previous hypotheses on .

The computation of the aforementioned probabilities relied on the predictive distribution in the embedding space, given previously observed embeddings. Since both changepoint and growth probabilities corresponded to different hypotheses on , it was imperative to have a unique predictive distribution congruent with each hypothesis. Each predictive distribution took into account a certain number of the most recently observed embeddings as dictated by the hypothesis on . At each time step, the current embedding, ${\varvec{o}}({}^{\Downarrow }{}{t_{k}})$, was presented to each of these predictive distributions. The resulting set of predictive probabilities then played a pivotal role in guiding the Bayesian inference, aiding in the determination of which hypotheses regarding  held greater plausibility and which ones were comparatively less tenable. Consequently, the more plausible predictive distributions effectively captured the evolving spatial distribution of the embedding time series.

However, the Bayesian inference did not operate on the predictive distributions directly. Instead, each predictive distribution’s (mean and precision) hyperparameters were implicitly defined by constructing a conjugate probability distribution over both hyperparameters simultaneously. This conjugate distribution was also modelled as a multivariate process. The role of the conjugate model was to communicate the prior belief about the spatial distribution of the embeddings to the Bayesian inference, before the inference of .

A base conjugate model was constructed before observing any embeddings and utilised at ${}^{\Downarrow }{}{t_0}$. This same base model also applied to the changepoint hypothesis at each time step, since this particular hypothesis assumed . However, for the other “growth” hypotheses for , other conjugate models were incrementally created using Bayesian inference, serving as modified versions of the original base model given the observed embeddings dictated by their respective hypotheses.

The construction of the base conjugate model depended on which dimension reduction method was employed in the second stage of the framework. If UMAP was used, the conjugate model’s probability distribution was set to be a *non-informative prior distribution* due to the unsupervised nature of the method and the ambiguous embedding topology evident in its egg-like structure. The non-informative prior was intentionally designed to spread widely (almost flat) over the *mean-precision hyperparameter space* such that no particular combination was favoured in any way. In such a case, the Bayesian inference was instructed to objectively construct the posterior distribution over the hyperparameters prominently based on the observed UMAP embeddings.

In contrast, if ADR was used for dimension reduction in the second stage of the framework, it was feasible to assign an *informative prior distribution* to the conjugate model, leveraging the consistent geometrical configuration of the ADR embeddings. The informative prior guided the Bayesian inference on the likely topology of the ADR embeddings, rendering higher probability density to the plausible mean-precision combinations in the hyperparameter space.

The implementation of the Bayesian inference method outlined above had a distinct difference from that presented in the original paper^41^. The original paper employed univariate predictive distributions and demonstrated their use in the inference of either the mean or the precision of uni-dimensional time series. However, this stage of the KIDS framework adapted the original method to support the multivariate inference of both the evolving mean vector and precision matrix for the three-dimensional embedding time series. A thorough delineation of the implementation can be found in Supplementary Methods titled “Bayesian inference of current segment run length”.

The Bayesian inference method provided a robust mechanism to discern the duration of periods of sustained postural inactivity within the embedding time series. With this foundation, the next methodological step made a critical transition in transforming the multitude of outcomes encoded in the posterior distribution over  into a singular, most probable estimate at each time step.

##### Probabilistic point estimation (Step 2)

 The aforementioned Bayesian inference method produced a posterior distribution over , conditioned on the observed embeddings until time step ${}^{\Downarrow }{}{t_k}$. It is important to emphasise that  was treated as a discrete random variable in this context. Each possible value that this variable could assume under the posterior distribution corresponded to a unique hypothesis. The objective then shifted towards pinpointing a singular, most probable estimate for . Consequently, the use of *probabilistic point estimation* was proposed to determine the best estimate, denoted as , according to some probabilistic sense^42^. To this end, the *least mean squares* (LMS) estimator was employed to determine  by minimising the *mean squared error*, conditioned on the sequence of observed embeddings, ${\varvec{o}}({}^{\Downarrow }{}{t_{1:k}})$:4

Utilising the LMS estimator in Eq. (4) brought forth the advantage of striking an optimal trade-off between bias and variance. This methodological step culminated in producing a vector, encapsulating $(T+1)$ LMS estimates of  throughout the time series.

However, a subtle artefact which surfaced in the posterior distribution over  during specific posture transitions had an effect on the computed LMS estimates. Notably, while the majority of posture transitions involved an immediate surge in the changepoint probability in tandem with the commencement of the transition motion, certain instances of posture transitions were associated with a slightly slower inference response. In these instances, the “growth” hypothesis probability dwindled over time, and this wane was offset by a corresponding rise in the changepoint probability spanning a few time steps. Such an artefact led the LMS estimates to exhibit a gradual reset to zero over a few time steps from the actual commencement of the posture transition motion. Further analysis revealed two consequences of this behaviour in regard to the performance of the KIDS framework. Firstly, the magnitude of change in  was diluted across the transitions that had this artefact, making changepoints harder to be detected later within the framework. Secondly, it distorted the posterior distribution over  nearer to the detected changepoints, which caused significant underestimations in the duration of postural inactivity for these instances, even if their corresponding changepoints were detected. These observations motivated the employment of an “enhancement step”, delineated in the ensuing methodological step, to rectify these artefacts and to ensure the fidelity of the proposed framework outcomes. The added value of this enhancement step was identified through an ablation analysis at the end of “Methods” section, encompassing the four variants of the KIDS framework.

##### Enhancement of the LMS estimates (Step 3)

 The enhancement step was incorporated into the KIDS framework to address the aforementioned inference artefact: the progressive reset of  to zero during specific posture transitions. Addressing this artefact was imperative for the subsequent steps within the framework in order to promote a robust detection of posture changepoints and prevent inadvertent underestimation of the durations of postural inactivity periods.

To counteract the effect of the inference artefact, a moving filter, spanning a width of three time steps, was proposed. As this artefact was characterised by consecutive progressive drops in the value of  over a few time steps, the primary objective of this filter was to reinforce the magnitude of change in  during the transitions marked by the artefact. The realisation of this objective culminated in a new sequence of “enhanced LMS estimates”, denoted by . Here, the strategy was to amalgamate the consecutive drops in the LMS estimates (spanning multiple time steps) into a single pronounced dip between adjacent time steps, described mathematically in Eq. (5):5

The following illustrative scenario elucidates the operation of the moving filter. If there were two sequential drops in the LMS estimates, characterised by values , , and , the resultant enhanced sequence would be manifested as , , and . By employing this moving filter, the sequential drops were translated to one sheer dip of 30 time steps between  and . Consequently, the changepoint event was accentuated, rendering it significantly more conspicuous for the ensuing changepoint detection logic. Notably, while the changepoint was discerned at , a single time step post the initiation of the posture transition motion, the proposed filter crucially obviated the larger risk of completely overlooking the changepoint event.

##### Changepoint detection logic (Step 4)

 Embedded within the enhanced LMS estimates was invaluable information concerning the durations of postural inactivity and the temporal locations of sleep posture changepoints. The quintessential aim of this ultimate step was to pinpoint the exact time steps wherein  exhibited a pronounced dip in its value. A changepoint detection logic was meticulously crafted to accomplish this aim. This logic produced a timeline of binary decisions, with positive outcomes signifying the detected changepoints. Subsequently, by leveraging the time step corresponding to the changepoint and the value of the enhanced LMS estimate that immediately preceded it, the start and end timestamps of each postural inactivity period were determinable.

The operation of the changepoint detection logic was bifurcated into two consecutive steps. The first step aimed at the exhaustive identification of all changepoints, whilst the second step equipped end-users, such as reporting clinicians, with the flexibility to selectively discard changepoints of insignificant relevance to their practice.

The first step entailed a critical stipulation: *the decision-making process should be invariant to the scale of*, meaning that the dips from  and  ought to be treated equivalently, regardless of whether $\zeta _b \gg \zeta _a$. A mere thresholding of the magnitude drop, , in the linear scale would fail to meet the scale-invariance criterion. A more viable proposition entailed the implementation of the threshold on the logarithmic scale of , thus grounding the decision-making of the changepoint detection logic on percent change or multiplicative factors. A reference drop of $\log _{10} 2 \approx 0.3$ on the logarithmic scale corresponded to a 50% decrease in  in the linear scale. Since this halving borderline was reasonably poised to provide the sought binary decisions, it was incorporated into the changepoint detection logic as the triggering criterion for detecting posture changepoints. Consequently, a changepoint was flagged when the consecutive difference expression, , surpassed the threshold of 0.3.

Subsequent to the exhaustive identification of changepoints in the first step, the second step introduced an additional layer of decision-making for selective reporting of changepoints and their respective postural inactivity segments. For instance, in sleep medicine, provocative sleep postures could bring adverse health implications when sustained for long periods. Therefore, it would be more reasonable to offer the capability of selecting only the changepoints that were associated with sufficiently long periods of inactivity. Additionally, from a purely methodological perspective, transition motions in between sleep postures were found to repeatedly trigger multiple changepoints as the human participant made few posture adjustments before settling into the new posture. Motivated by the irrelevance of these transitionary posture adjustments to the main problems addressed by the KIDS framework, which were primarily oriented about sleep posture changes and sustained postural inactivity, the logic was extended to discard changepoints for which their preceding enhanced LMS estimate fell short of twenty time steps, a condition supported by histograms of the differences in the enhanced LMS estimate before and after all detected changepoints across assorted participant datasets. An example of such a histogram can be found online in Supplementary Fig. S6.

As mentioned in “Discussion” section, a potentially “weaker” changepoint event might occur in the rare case where the wrist joint orientation does not change significantly across two consecutive sleep postures, especially if the intermediate posture transition was brief. While this particular case only happened once, the changepoint was detected nevertheless. However, following this weak changepoint event, it was found that the LMS estimates started to increase linearly, but not from zero. Instead, they increased from the last LMS estimate before the changepoint. This rare pattern was caused mainly by the brief transition in posture, which, in return, activated an auto-corrective behaviour within the Bayesian inference method. This behaviour led the method to mistakenly perceive the embedding at the changepoint as an anomaly within a larger coherent segment of embeddings, i.e. a larger inactivity segment. While this auto-correction does not affect the temporal segmentation of the inactivity segment terminated at the weak changepoint event, it would lead to an overestimation of the duration of the subsequent inactivity segment. To prevent this overestimation, an upper limit was imposed on the duration of postural inactivity, equivalent to the elapsed time steps since the previous changepoint. This adjustment ensured that the duration of postural inactivity was not overestimated.

With this step, the strategic plan devised within the ultimate stage of the KIDS framework was concluded. Within the overarching Methods Section, a multitude of techniques was delineated which included design choices, rendering different variants of the proposed framework. A summary of all hyperparameters employed in the framework can be found in Supplementary Table S1. The ensuing section compares the performance of these variants and develops a good understanding of the added value of the design choices that were made.

#### Ablation analysis

The KIDS framework, which spanned three pivotal stages, comprised several methods. While the foundations of these methods were grounded in logical principles, it was nevertheless important to recognise certain design choices that emerged from two main sources of variation.

The first source of variation stemmed from a design choice within the second stage of the KIDS framework, specifically during the dimension reduction step. Therein, two distinct methods of dimension reduction were examined: UMAP and ADR. While ADR rendered better embedding topology in comparison to that of UMAP, the qualitative visual appraisal alone did not inform the implications this design choice would have for the performance of the framework.

The second source of variation arose from the framework’s third stage, wherein an enhancement step was introduced with the objective of enhancing the fidelity of the LMS estimates derived from the inferred Bayesian posterior, particularly during specific transitionary motions in between sleep postures. While this step facilitated the refinement of estimated durations of postural inactivity, further evidence was still required to validate its positive contribution to the overall performance of the framework.

**Table 2:** The comparative performance metrics for the four variants of the KIDS framework across two problem domains.

Performance metric	KIDS (I)	KIDS (II)	KIDS (III)	KIDS (IV)
Problem domain 1: sleep posture change detection				
F$_{1}$-score	0.91	0.90	0.97	**0.99**
Se	0.93	0.91	0.95	**1.00**
PPV	0.89	0.89	**0.98**	**0.98**
Problem domain 2: temporal segmentation of postural inactivity				
R	0.63	0.78	0.81	**0.96**

The metrics were averaged over five participants (P1–P5). The variants encompassed: KIDS with UMAP sans the enhancement step (I), KIDS with UMAP coupled with the enhancement step (II), KIDS with ADR excluding the enhancement step (III), and KIDS amalgamating ADR with the enhancement step (IV). The metrics unveiled insights into the efficacy of the variants in addressing sleep posture change detection (Problem Domain 1) and the temporal segmentation of postural inactivity (Problem Domain 2). Notably, the metrics demonstrate the superiority of the simultaneous use of ADR and the enhancement step, as evidenced by the bolded scores indicating the highest performance across each metric.

In light of these methodological variations, an ablation analysis was conducted to develop a more profound comprehension of the contributions of these two constituent design choices, namely the transition from UMAP to ADR and the incorporation of the enhancement step, to the framework. This ablation analysis was structured to critically assess the KIDS framework both in the presence and absence of these design choices, hence casting light on their intrinsic effects on its performance. Four variants of the framework, labelled from I to IV, evolved from this analysis: KIDS with UMAP sans the enhancement step (I), KIDS with UMAP coupled with the enhancement step (II), KIDS with ADR excluding the enhancement step (III), and KIDS amalgamating ADR with the enhancement step (IV).

The performance metrics for all variants (I–IV), aggregated across the five human participants (P1–P5), are succinctly captured in Table 2. These metrics provided insights into the variants across the two interrelated problem domains: the sleep posture change detection (Problem Domain 1) and the temporal segmentation of postural inactivity (Problem Domain 2). An important finding was the superior performance of ADR-based variants, namely KIDS (III) and (IV), over their UMAP-based counterparts across both problem domains. Furthermore, it was confirmed that the synergetic use of ADR with the enhancement step indeed rendered the best-performing variant of the framework not only from a (qualitative) visualisation perspective but also in terms of the (quantitative) performance metrics. In contrast, the least effective variants were contingent on the problem domain, with KIDS (I) and (II) yielding the least performance metrics in the second and first problem domains, respectively. Besides these metrics, Supplementary Figs. S3–S6 provide visual insights into the performance of all four variants, demonstrated on the dataset of participant (P1).

**Table 3:** The net change in the performance metrics across five ablation experiments (AE), each of which contrasted a unique pair from the four variants of the KIDS framework.

Ablation experiment	KIDS (I)	KIDS (II)	KIDS (III)	KIDS (IV)	Problem domain	Performance metric	Mean net change
AE 1	$\checkmark$	–	$\checkmark$	–	1	F$_{1}$-score	0.06
						Se	0.06
						PPV	0.09
					2	R	0.18
AE 2	–	$\checkmark$	–	$\checkmark$	1	F$_{1}$-score	0.09
						Se	0.09
						PPV	0.09
					2	R	0.18
AE 3	$\checkmark$	$\checkmark$	–	–	1	F$_{1}$-score	(−) 0.01
						Se	(−) 0.02
						PPV	0.00
					2	R	0.15
AE 4	–	–	$\checkmark$	$\checkmark$	1	F$_{1}$-score	0.02
						Se	0.05
						PPV	0.00
					2	R	0.15
AE 5	$\checkmark$	–	–	$\checkmark$	1	F$_{1}$-score	0.08
						Se	0.07
						PPV	0.17
					2	R	0.33

The net change in the metrics was averaged over five participants (P1–P5). The variants encompassed: KIDS with UMAP sans the enhancement step (I), KIDS with UMAP coupled with the enhancement step (II), KIDS with ADR excluding the enhancement step (III), and KIDS amalgamating ADR with the enhancement step (IV). Each experiment served to discern the value attributed to the design choices that led to the variants. The performance metrics were categorised according to the problem domains: sleep posture change detection (Problem Domain 1) and temporal segmentation of postural inactivity (Problem Domain 2). The ablation analysis revealed three pivotal observations: (1) the consistently superior performance rendered by ADR-based variants regardless of the incorporation of the enhancement step (AE 1 and AE 2), (2) the pronounced benefit of the enhancement step particularly for ADR-based variants (AE 3 and AE 4), and (3) the amalgamated gains accrued from the simultaneous utilisation of ADR and the enhancement step exceeded the individual advantages offered by either one of them (AE 5).

For a more granulated perspective on the value contributed by each design choice, Table 3 elucidates the mean net change in performance metrics across five strategically devised ablation experiments (AE), each considering a unique pair of variants. AE 1 and AE 2 endeavoured to quantify the contribution of switching from UMAP to ADR in the dimension reduction stage, under scenarios with and without the enhancement step, respectively. Both experiments showcased that mean net change in the metrics was clearly in favour of ADR across both problem domains, irrespective of the enhancement step’s deployment. Moving on to AE 3 and AE 4, both experiments were conceived to gauge the effect of the enhancement step on the variants based on UMAP and ADR respectively. It was found that the incorporation of the enhancement step was apparently more rewarding for ADR-based variants in comparison to their UMAP-based counterparts. Specifically, while the enhancement step augmented the performance across both domains for KIDS (IV), it nonetheless bolstered only the segmentation performance for KIDS (II). Lastly, AE 5 juxtaposed KIDS (IV) against KIDS (I) to discern the amalgamated benefits of transitioning from UMAP to ADR and the concurrent inclusion of the enhancement step. The resultant analysis underscored the substantially high performance of KIDS (IV), evidenced by a mean net improvement of 0.08 in the F$_{1}$-score and 0.33 in the R coefficient, compared to KIDS (I).

Besides the four-variant comparison outlined above, an additional ablation study was conducted to evaluate the effect of bypassing the dimension reduction step entirely within the second stage of the KIDS framework. This ablation study was important to explore whether the value of dimension reduction extended beyond intuitive visualisation and efficient computational performance. In the absence of low-dimensional embeddings, the downsampled hand-to-forearm orientations were fed into the third stage of the framework in the form of quaternions. Consequently, the informative prior distribution was adapted to the characteristics of the quaternion space. The outcome of this study was a substantial decline in the performance metrics across both problem domains, yielding 0.34 and 0.20 for the F$_{1}$-score and correlation coefficient, respectively, averaged over the five participant datasets. Notably, further implications of omitting dimension reduction include a 25% increase in data storage requirements and a 3.37% reduction in computational efficiency.

### Supplementary Information

Supplementary Information.

## Data Availability

The datasets generated and/or analysed during the current study are available in the *Liverpool Data Catalogue* repository^43^, DOI:10.17638/datacat.liverpool.ac.uk.
