# Supplementary material for: An interpretable framework for sleep posture change detection and postural inactivity segmentation using wrist kinematics
Source: Sci Rep. 2023 Oct 21;13:18027. doi: 10.1038/s41598-023-44567-9 (PMC10590424; doi:10.1038/s41598-023-44567-9)
Supplement: Supplementary file 1 — Supplementary Information. [file 41598_2023_44567_MOESM1_ESM.pdf]

An Interpretable Framework for Sleep Posture Change Detection and Postural Inactivity Segmentation Using Wrist Kinematics

Omar Elnaggar[1], Roselina Arelhi[2], Frans Coenen[3], Andrew Hopkinson[4], Lyndon Mason[5,6], and Paolo Paoletti[1,*]

[1]School of Engineering, University of Liverpool, Liverpool L69 3GH, United Kingdom
[2]Faculty of Engineering, University of Sheffield, Sheffield S1 3JD, United Kingdom
[3]School of Electrical Engineering, Electronics and Computer Science, University of Liverpool, Liverpool L69 3BX, United Kingdom
[4]School of Psychology,University of Liverpool, Liverpool L69 7ZA, United Kingdom
[5]School of Medicine, University of Liverpool, Liverpool L69 3GE, United Kingdom
[6]Department of Trauma and Orthopaedics, Liverpool University Hospitals NHS Foundation Trust, Liverpool L9 7AL, United Kingdom
[*]P.Paoletti@liverpool.ac.uk

Supplementary Information

Supplementary Methods - Computer Graphics Pipeline

Even though the axis-angle representation is intuitive, its four dimensions do not facilitate easy visualisation of joint orientation time series. Therefore, UMAP and analytical dimension reduction were used to reduce the dimensions to three. With fewer dimensions, the computational cost of subsequent Bayesian inference was lowered. This section is focused on the generation of synthetic orientation data essential for the UMAP-based dimension reduction.

Sensor-measured joint orientations are typically limited in size and do not cover the entire axis-angle orientation space due to anatomical constraints. For these reasons, forcing the UMAP to learn the data manifold of $\boldsymbol{x}$ from sensor measurements does not guarantee accurate manifold modelling and may suffer from mapping discontinuities. Therefore, this paper proposes the generation of a synthetic axis-angle orientation dataset better suited for manifold learning by UMAP. Leveraging a *computer graphics* (CG) pipeline, synthetic axes of rotation evenly distributed over the surface of a unit sphere were obtained. Then, an equidistant sequence of scalar angles of rotation was created. Finally, the synthetic orientation dataset was formed by exhausting all possible combinations between the axes and angles of rotation.

The CG pipeline aims at the procedural 3D modelling of a unit sphere with evenly-distributed vertices (axes of rotation). As depicted in Fig. S1, the pipeline generally consisted of two stages: (i) the construction of a unit cube with a structured triangular mesh, and (ii) the projection of the cube vertices onto the surface of a unit sphere. All procedural 3D modelling was implemented in the C# programming language and realised in Unity© (Unity Technologies Inc., California, US).

The first stage of the CG pipeline involves procedural construction of the six faces making the unit cube. Each quadrilateral face is defined by a *surface normal vector*, $\vec{n}_{face}$, and a predefined *mesh resolution*, Υ, such that Υ is the number of vertices along any side of the quadrilateral face. Given these definitions, the vertex grid of the face was constructed with Υ^2 vertices, each assigned a unique identifier index. The cube faces are all procedurally constructed using $\Upsilon = 15$ and $\vec{n}_{face}$ being the six orthonormal directions of Unity's world coordinate system, which yields a total of 1,350 vertices defining the cube.

Next, the cube vertices need to be projected onto the surface of a unit sphere to obtain the synthetic axes of rotation. To this end, two approaches, based on *Euclidean normalisation* and *ellipsoidal projection* respectively, were employed and compared.

First Approach: Projection of Cube Vertices with Euclidean Normalisation

Let $\boldsymbol{v}_c$ denote an arbitrary vertex on the unit cube, with individual coordinates (x_c, y_c, z_c). This approach projects $\boldsymbol{v}_c$ onto the surface of a unit sphere through the normalisation of $\boldsymbol{v}_c$ by its *Euclidean norm* as defined in Eq. (S1).

$$^{\text{euclid}}\boldsymbol{v}_s = \frac{1}{\sqrt{x_c^2 + y_c^2 + z_c^2}} \cdot \begin{bmatrix} x_c & y_c & z_c \end{bmatrix} \tag{S1}$$

The result of this projection is displayed in Fig. S1a, which clearly shows projected vertices clumped together at the corners of the faces than in the centres. Eventually, this projection would produce non-uniform samples over the data manifold in the

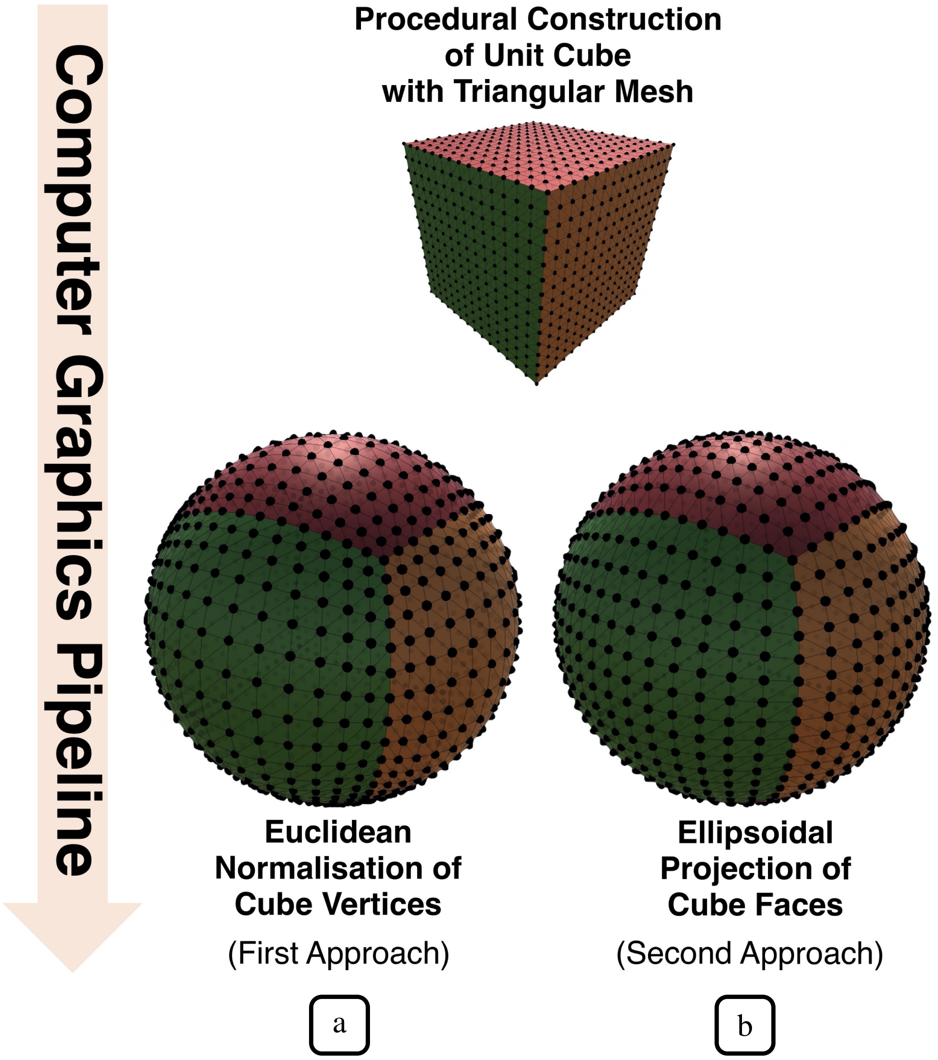

Figure S1. The computer graphics pipeline used for the generation of synthetic axes of rotation.

orientation space, and could potentially cause unstable dimension reduction. Therefore, a second approach was developed to address this projection artefact.

Second Approach: Ellipsoidal Projection of Cube Faces

To obtain better distributed vertices, this approach projects constant-x, constant-y and constant-z square faces, which are the basic building geometries of a cube. The principal idea is to project each face onto an ellipsoid, and the stitched face ellipsoidal projections together make a unit sphere. Suppose a generic face vertex, $\boldsymbol{v}_f$, defined as

$$\boldsymbol{v}_f = \{(x_f, y_f, z_f) \mid x_f, y_f, z_f \in [-1, 1]\} \tag{S2}$$

where either x_f, y_f or z_f is fixed at some constant value between -1 to 1, depending on the face orientation defined by its surface normal vector. Mathematically, the ellipsoidal projection aims to map face vertices $\boldsymbol{v}_f$ to vertices $\boldsymbol{v}_e$ on a 3D ellipsoid such that the point of intersection with the coordinate axis remain unchanged, i.e. $\boldsymbol{v}_e = \boldsymbol{v}_f$ at the centre of the face, and the face vertices are more normalised the closer they are to the corners of the face. For this requirement to hold true, the ellipsoid equation for some constant-x_f face, as an example, must be

$$1 = \frac{x_e^2}{x_f^2} + \frac{y_e^2}{b_e^2} + \frac{z_e^2}{c_e^2} \tag{S3}$$

where b_e and c_e are constants, and $b_e = c_e$ due to symmetry about $x_e - y_e$ and $x_e - z_e$ planes. As x_f is varied from -1 to 1, the ellipsoid in Eq. (S3) make two intersection curves with the unit sphere, one curve for each of the upper and lower hemispheres.

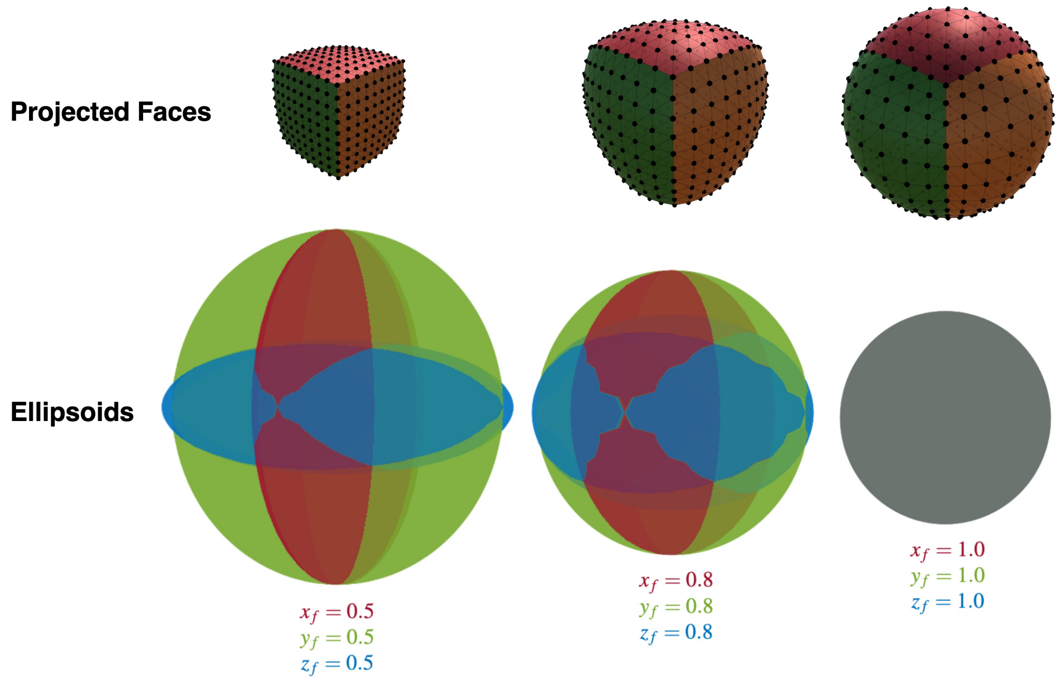

Figure S2. The effect of cube face displacement on the ellipsoidal projection.

Any known point along these intersection curves can be used to determine b_e and c_e. The upper intersection curve meets the $x_e - y_e$ plane at

$$\boldsymbol{v}_e = \begin{bmatrix} x_e & y_e & z_e \end{bmatrix} = \begin{bmatrix} \frac{1}{\sqrt{2}}\, x_f & \frac{1}{\sqrt{2}}\sqrt{1-\frac{1}{2}\, x_f^2} & \frac{1}{\sqrt{2}}\sqrt{1-\frac{1}{2}\, x_f^2} \end{bmatrix} \tag{S4}$$

Substituting Eq. (S4) into Eq. (S3) yields $b_e = c_e = 2 - x_f^2$. Hence, the ellipsoid for the constant-x face becomes

$$1 = \frac{x_e^2}{x_f^2} + \frac{y_e^2}{2-x_f^2} + \frac{z_e^2}{2-x_f^2} \tag{S5}$$

By symmetry, the constant-y and constant-z faces are similarly mapped to the ellipsoids defined by Eqs. (S6) and (S7) respectively.

$$1 = \frac{x_e^2}{2-y_f^2} + \frac{y_e^2}{y_f^2} + \frac{z_e^2}{2-y_f^2} \tag{S6}$$

$$1 = \frac{x_e^2}{2-z_f^2} + \frac{y_e^2}{2-z_f^2} + \frac{z_e^2}{z_f^2} \tag{S7}$$

Then, the system of equations Eqs. (S5) to (S7) are simultaneously solved for x_e, y_e and z_e to obtain the general expression for the ellipsoidal projection, $\boldsymbol{v}_e$

$$\boldsymbol{v}_e = \begin{bmatrix} x_e & y_e & z_e \end{bmatrix} = \begin{bmatrix} x_f\sqrt{1-\frac{1}{2}\, y_f^2-\frac{1}{2}\, z_f^2+\frac{1}{3}\, y_f^2 z_f^2} & y_f\sqrt{1-\frac{1}{2}\, x_f^2-\frac{1}{2}\, z_f^2+\frac{1}{3}\, x_f^2 z_f^2} & z_f\sqrt{1-\frac{1}{2}\, x_f^2-\frac{1}{2}\, y_f^2+\frac{1}{3}\, x_f^2 y_f^2} \end{bmatrix} \tag{S8}$$

Fig. S2 shows the ellipsoids for constant-x, constant-y and constant-z faces at different displacements from the origin of the Cartesian coordinate system. As x_f, y_f and z_f converge to 1.0, their corresponding ellipsoids converge in 3D shape to a unit sphere. This specific case of convergence applies to a cube with a side length of 1 unit, where the ellipsoidal projections of all six faces form a complete sphere when stitched together. Therefore, a better distributed vertex projection ${}^{\text{ellips}}\boldsymbol{v}_s$ (see Fig. S1b) of the cube vertices onto the surface of a unit sphere was obtained using Eq. (S8).

Each projected vertex on the surface of the sphere represents a candidate Cartesian axis of rotation. All 1,350 axes of rotation were written to a *comma-separated values* (CSV) file for subsequent import into MATLAB© (The MathWorks, Massachusetts, US). Therein, an orientation dataset generator script concatenated each synthetic axis of rotation with each angle of rotation from the closed set $\{\frac{36}{36}\pi, \frac{35}{36}\pi, \frac{34}{36}\pi, \ldots, \frac{1}{36}\pi\}$. The output synthetic axis-angle dataset contained a total of 48,600 orientations.

Supplementary Methods - Bayesian Inference of Current Segment Run Length

Based on the original paper[1], the general mathematical expression for the joint probability can be written as:

$$P(\mathcal{R}_k,\, \boldsymbol{o}({}^{\Downarrow}t_{1:k})) = \sum_{\mathcal{R}_{k-1}} \underbrace{P(\boldsymbol{o}({}^{\Downarrow}t_k) \mid \mathcal{R}_k,\, \boldsymbol{o}({}^{\Downarrow}t_{1:k-1}))}_{\text{Predictive Term}} \quad \underbrace{P(\mathcal{R}_k \mid \mathcal{R}_{k-1})}_{\text{Changepoint Prior}} \quad \underbrace{P(\mathcal{R}_{k-1},\, \boldsymbol{o}({}^{\Downarrow}t_{1:k-1}))}_{\text{Recursive Term}} \tag{S9}$$

The Predictive Term. It is also referred to as the *posterior predictive term* because it predicts the next embedding given the previously observed embeddings and $\mathcal{R}_k$. Thus, if a hypothesis states $\mathcal{R}_k = \zeta$, only embeddings within the past ζ time steps should contribute to the predictive term:

$$P(\boldsymbol{o}({}^{\Downarrow}t_k) \mid \mathcal{R}_k = \zeta,\, \boldsymbol{o}({}^{\Downarrow}t_{1:k-1})) = P(\boldsymbol{o}({}^{\Downarrow}t_k) \mid \mathcal{R}_k = \zeta,\, \boldsymbol{o}({}^{\Downarrow}t_{k-\zeta:k-1})) \tag{S10}$$

In this paper, it is assumed that $\mathcal{O}({}^{\Downarrow}\boldsymbol{t})$ is continuously distributed with unknown mean vector $\boldsymbol{\mu}_{\mathcal{O}} \in \mathbb{R}^3$ and precision matrix $\boldsymbol{\lambda}_{\mathcal{O}} \in \mathbb{R}^{3\times 3}$. Additionally, a likelihood model, $P_l(\boldsymbol{o}({}^{\Downarrow}t_k) \mid \boldsymbol{\eta})$, is defined with model parameters $\boldsymbol{\eta} = \{\boldsymbol{\mu}_{\mathcal{O}},\, \boldsymbol{\lambda}_{\mathcal{O}}\}$. To compute the predictive term, Eq. (S11) first finds the posterior distribution, $P(\boldsymbol{\eta} \mid \mathcal{R}_k = \zeta,\, \boldsymbol{o}({}^{\Downarrow}t_{k-\zeta:k-1}))$, then marginalises $\boldsymbol{\eta}$, conditional on $\mathcal{R}_k$. Hence, the predictive term becomes the posterior predictive distribution:

$$P(\boldsymbol{o}({}^{\Downarrow}t_k) \mid \mathcal{R}_k = \zeta,\, \boldsymbol{o}({}^{\Downarrow}t_{k-\zeta:k-1})) = \int_{\boldsymbol{\eta}} P_l(\boldsymbol{o}({}^{\Downarrow}t_k) \mid \boldsymbol{\eta}) \quad P(\boldsymbol{\eta} \mid \mathcal{R}_k = \zeta,\, \boldsymbol{o}({}^{\Downarrow}t_{k-\zeta:k-1})) \quad d\boldsymbol{\eta} \tag{S11}$$

The posterior distribution can be re-written as $P(\boldsymbol{\eta} \mid \mathcal{D},\, \boldsymbol{\alpha})$, where $\mathcal{D} = \boldsymbol{o}({}^{\Downarrow}t_{k-\zeta:k-1})$ and $\boldsymbol{\alpha}$ denotes the parameters of the prior distribution, $P(\boldsymbol{\eta} \mid \boldsymbol{\alpha})$. Using Bayes' theorem, conditional probability and chain rule, $P(\boldsymbol{\eta} \mid \mathcal{D},\, \boldsymbol{\alpha})$ can be expanded as follows:

$$P(\boldsymbol{\eta} \mid \mathcal{D},\, \boldsymbol{\alpha}) = \frac{P_l(\mathcal{D} \mid \boldsymbol{\eta},\, \boldsymbol{\alpha})\, P(\boldsymbol{\eta} \mid \boldsymbol{\alpha})}{P(\mathcal{D} \mid \boldsymbol{\alpha})} \tag{S12}$$

However, computing the integral in Eq. (S11) is intractable. Fortunately, *conjugate-exponential models*[2] provide an efficient solution to this problem. The concept of conjugacy allows for the derivation of a closed form expression as a function of $\boldsymbol{\alpha}$ without the need for integration. Hence, given a likelihood model $P_l(\mathcal{D} \mid \boldsymbol{\eta},\, \boldsymbol{\alpha})$, its conjugate prior model can be leveraged to simplify the posterior distribution to:

$$P(\boldsymbol{\eta} \mid \mathcal{D},\, \boldsymbol{\alpha}) = P(\boldsymbol{\eta} \mid \boldsymbol{\alpha}') \tag{S13}$$

where both the prior and posterior distributions belong to the same distribution family with parameters $\boldsymbol{\alpha}$ and $\boldsymbol{\alpha}'$ respectively.

Since both $\boldsymbol{\mu}_{\mathcal{O}}$ and $\boldsymbol{\lambda}_{\mathcal{O}}$ are inferred, the likelihood model follows a *multivariate Gaussian distribution*, for which the natural conjugate prior is the *Normal-Wishart distribution* with quadruple parameterisation $\boldsymbol{\alpha} = \{\boldsymbol{\mu}_\alpha,\, \kappa_\alpha,\, \nu_\alpha,\, \boldsymbol{\Sigma}_\alpha\}$,

$$\begin{aligned} P(\boldsymbol{\eta} \mid \boldsymbol{\alpha}) &= \mathcal{NW}(\boldsymbol{\eta} \mid \boldsymbol{\alpha}) \\ &= \mathcal{N}(\boldsymbol{\mu}_{\mathcal{O}} \mid \boldsymbol{\mu}_\alpha,\, (\kappa_\alpha\, \boldsymbol{\lambda}_{\mathcal{O}})^{-1}) \quad \mathcal{W}(\boldsymbol{\lambda}_{\mathcal{O}} \mid \boldsymbol{\Sigma}_\alpha,\, \nu_\alpha) \end{aligned} \tag{S14}$$

where $\boldsymbol{\mu}_\alpha \in \mathbb{R}^3$ and $\kappa_\alpha \in \mathbb{R}$ are the mean vector and the covariance scale coefficient, respectively, of the normal distribution, and $\nu_\alpha \in \mathbb{R}$ and $\boldsymbol{\Sigma}_\alpha \in \mathbb{R}^{3\times 3}$ are the number of degrees of freedom and the scale matrix, respectively, of the Wishart distribution.

Since $P(\boldsymbol{\eta} \mid \boldsymbol{\alpha})$ is a conjugate prior, then $P(\boldsymbol{\eta} \mid \mathcal{D},\, \boldsymbol{\alpha})$ turns out to be a Normal-Wishart distribution too. Substituting $P(\boldsymbol{\eta} \mid \mathcal{D},\, \boldsymbol{\alpha})$ into Eq. (S11), it can be shown that the posterior predictive distribution follows a *Student's t-distribution* with $(\nu'_\alpha - 2)$ degrees of freedom:

$$\begin{aligned} P(\boldsymbol{o}({}^{\Downarrow}t_k) \mid \mathcal{D},\, \boldsymbol{\alpha}) &= \int P_l(\boldsymbol{o}({}^{\Downarrow}t_k) \mid \boldsymbol{\eta}) \quad P(\boldsymbol{\eta} \mid \mathcal{D},\, \boldsymbol{\alpha}) \quad d\boldsymbol{\eta} \\ &= \mathcal{T}_{\nu'_\alpha - 2}(\boldsymbol{o}({}^{\Downarrow}t_k) \mid \boldsymbol{\mu}'_\alpha,\, \boldsymbol{\Sigma}'_\alpha(\kappa'_\alpha + 1){\kappa'_\alpha}^{-1}(\nu'_\alpha - 2)^{-1}) \end{aligned} \tag{S15}$$

where $\boldsymbol{\mu}'_\alpha$ and $\boldsymbol{\Sigma}'_\alpha(\kappa'_\alpha + 1){\kappa'_\alpha}^{-1}(\nu'_\alpha - 2)^{-1}$ are the predicted $\boldsymbol{\mu}_{\mathcal{O}}$ and $\boldsymbol{\lambda}_{\mathcal{O}}^{-1}$, respectively, of the posterior predictive distribution.

Setting the prior on preprocessed joint kinematic timeseries. The set of parameters $\boldsymbol{\alpha}(k,\zeta)$ are updated at each time step ${}^{\Downarrow}t_k$ and for each hypothesis $\mathcal{R}_k = \zeta$. The (non-)informative prior on $\boldsymbol{\eta}$ was defined by setting the initial parameters $\boldsymbol{\alpha}(0,0)$ of the prior model to the appropriate values. In the case of the Normal-Wishart distribution, the non-informative prior, $\boldsymbol{\alpha}(0,0)$, was defined with $\boldsymbol{\mu}_\alpha(0,0) = \begin{bmatrix}10^{-4} & 10^{-4} & 10^{-4}\end{bmatrix}$, $\kappa_\alpha(0,0) = 10^{-4}$, $\nu_\alpha(0,0) = 4$ and any matrix $\boldsymbol{\Sigma}_\alpha(0,0)$ provided that its

determinant $|\boldsymbol{\Sigma}_\alpha(0,0)| = 0$. The non-informative prior is more suited for ${}^{\text{UMAP}}\boldsymbol{\mathcal{O}}$ due to the unconstrained embedding space of UMAP.

In contrast to UMAP, ADR has a constrained embedding space and known 3D embedding topology which facilitated the design of an informative prior. Visual inspection of the real preprocessed joint kinematics in the ADR embedding space informed the following settings: (1) $\boldsymbol{\mu}_{\mathcal{O}}$ has an expected value $E(\boldsymbol{\mu}_{\mathcal{O}}) = 0$ and an initial guess of $\boldsymbol{I}_{3\times3}$ for its covariance matrix, and (2) $\boldsymbol{\lambda}_{\mathcal{O}}$ has an expected value $\mathbb{E}[\boldsymbol{\lambda}_{\mathcal{O}}] = \nu_\alpha\,\boldsymbol{\Sigma}_\alpha = 20\,\boldsymbol{I}_{3\times3}$. Therefore, $\boldsymbol{\alpha}(0,0)$ was set such that $\boldsymbol{\mu}_\alpha(0,0) = \begin{bmatrix}10^{-4} & 10^{-4} & 10^{-4}\end{bmatrix}$, $\kappa_\alpha(0,0) = 1/20$, $\nu_\alpha(0,0) = 4$ and $\boldsymbol{\Sigma}_\alpha(0,0) = 5\,\boldsymbol{I}_{3\times3}$.

Updating the prior model parameters. At the end of each Bayesian inference step, the prior model parameters $\boldsymbol{\alpha}$ are updated to represent the new set of hypotheses on $\mathcal{R}_k$. At ${}^{\Downarrow}t_k$ and given an arbitrary hypothesis $\mathcal{R}_k = \zeta$, the prior model parameters denoted by $\boldsymbol{\alpha}(k,\zeta)$ are updated as follows:

$$\begin{aligned}
\boldsymbol{\mu}_\alpha(k,\zeta) &= \frac{\kappa_\alpha(0,0)\boldsymbol{\mu}_\alpha(0,0) + \zeta\,\bar{\boldsymbol{o}}_{k-\zeta+1:k}}{\kappa_\alpha(0,0)+\zeta}\\
\kappa_\alpha(k,\zeta) &= \kappa_\alpha(0,0) + \zeta\\
\nu_\alpha(k,\zeta) &= \nu_\alpha(0,0) + \zeta\\
\boldsymbol{\Sigma}_\alpha(k,\zeta) &= \boldsymbol{\Sigma}_\alpha(0,0) + \frac{\kappa_\alpha(0,0)\,\zeta}{\kappa_\alpha(0,0)+\zeta}(\boldsymbol{\mu}_\alpha(0,0) - \bar{\boldsymbol{o}}_{k-\zeta+1:k})\,(\boldsymbol{\mu}_\alpha(0,0) - \bar{\boldsymbol{o}}_{k-\zeta+1:k})^T +\\
&\qquad \sum_{i=1}^{\zeta}(\boldsymbol{o}({}^{\Downarrow}t_{k-i}) - \bar{\boldsymbol{o}}_{k-\zeta+1:k})\,(\boldsymbol{o}({}^{\Downarrow}t_{k-i}) - \bar{\boldsymbol{o}}_{k-\zeta+1:k})^T
\end{aligned} \tag{S16}$$

where $\bar{\boldsymbol{o}}_{k-\zeta+1:k}$ is the mean vector of the embeddings in $\boldsymbol{o}({}^{\Downarrow}t_{k-\zeta+1:k})$.

The Changepoint Prior. This term allows for encoding the prior knowledge on changepoints present in $\boldsymbol{\mathcal{O}}({}^{\Downarrow}\boldsymbol{t})$. The current segment run length $\mathcal{R}_k$ is is a discrete, nonnegative random variable this is subject to two possible outcomes at any given ${}^{\Downarrow}t_k$; either reset to zero upon a changepoint or an incremental growth by one. Thus, it was sensible to model $\mathcal{R}_k$ with a geometric distribution of success probability, p, where success corresponds to the growth of $\mathcal{R}_k$. Based on this modelling assumption, the changepoint prior term becomes

$$P(\mathcal{R}_k \mid \mathcal{R}_{k-1}) = \begin{cases} p, & \text{if } \mathcal{R}_k = 0.\\ 1-p, & \text{if } \mathcal{R}_k = \mathcal{R}_{k-1}+1.\\ 0, & \text{otherwise.}\end{cases} \tag{S17}$$

Using the changepoint prior in Eq. (S17), the joint probability term in Eq. (S9) can be further simplified. For growth transitions, it is redundant to sum over all possible $\mathcal{R}_{k-1}$ as the only valid run length is $\mathcal{R}_k = \mathcal{R}_{k-1}+1$. Substituting Eqs. (S13) and (S17) into Eq. (S9), the joint growth transition probability is defined as

$$P(\mathcal{R}_k,\,\boldsymbol{o}({}^{\Downarrow}t_{1:k})) = P(\boldsymbol{o}({}^{\Downarrow}t_k) \mid \boldsymbol{\alpha}(k-1,\mathcal{R}_{k-1}))\quad(1-p)\quad P(\mathcal{R}_{k-1},\,\boldsymbol{o}({}^{\Downarrow}t_{1:k-1})) \tag{S18}$$

For the changepoint transitions, the run length can be reset from any valid $\mathcal{R}_{k-1}$. Therefore, the summation over $\mathcal{R}_{k-1}$ is necessary,

$$P(\mathcal{R}_k,\,\boldsymbol{o}({}^{\Downarrow}t_{1:k})) = \sum_{\mathcal{R}_{k-1}} P(\boldsymbol{o}({}^{\Downarrow}t_k) \mid \boldsymbol{\alpha}(k-1,\mathcal{R}_{k-1}))\quad p\quad P(\mathcal{R}_{k-1},\,\boldsymbol{o}({}^{\Downarrow}t_{1:k-1})) \tag{S19}$$

After finding all joint probabilities $P(\mathcal{R}_k,\,\boldsymbol{o}({}^{\Downarrow}t_{1:k}))$ at ${}^{\Downarrow}t_k$, the posterior distribution on $\mathcal{R}_k$ was found using Bayes rule

$$P(\mathcal{R}_k \mid \boldsymbol{o}({}^{\Downarrow}t_{1:k})) = \frac{P(\mathcal{R}_k,\,\boldsymbol{o}({}^{\Downarrow}t_{1:k}))}{\sum_{\mathcal{R}_k} P(\mathcal{R}_k,\,\boldsymbol{o}({}^{\Downarrow}t_{1:k}))} \tag{S20}$$

where $\sum_{\mathcal{R}_k} P(\mathcal{R}_k,\,\boldsymbol{o}({}^{\Downarrow}t_{1:k}))$ is the normalising evidence.

Once the posterior distribution over $\mathcal{R}_k$ is determined $\forall k \in [0,T]$, this concludes the Bayesian inference of the current segment run length with $P(\mathcal{R}_k \mid \boldsymbol{o}({}^{\Downarrow}t_{1:k}))$ being a $(T+1)\times(T+1)$ matrix indexed by k and different $\mathcal{R}_k = \zeta$. The upper triangular part of this matrix contains all-zero probabilities since $\mathcal{R}_k \leq k$ at any ${}^{\Downarrow}t_k$.

References

1. Adams, R. P. & MacKay, D. J. C. Bayesian online changepoint detection. *arXiv preprint* DOI: 10.48550/arxiv.0710.3742 (2007).

2. Murphy, K. P. Conjugate bayesian analysis of the gaussian distribution. *Def* **1**, 1–29 (2007).

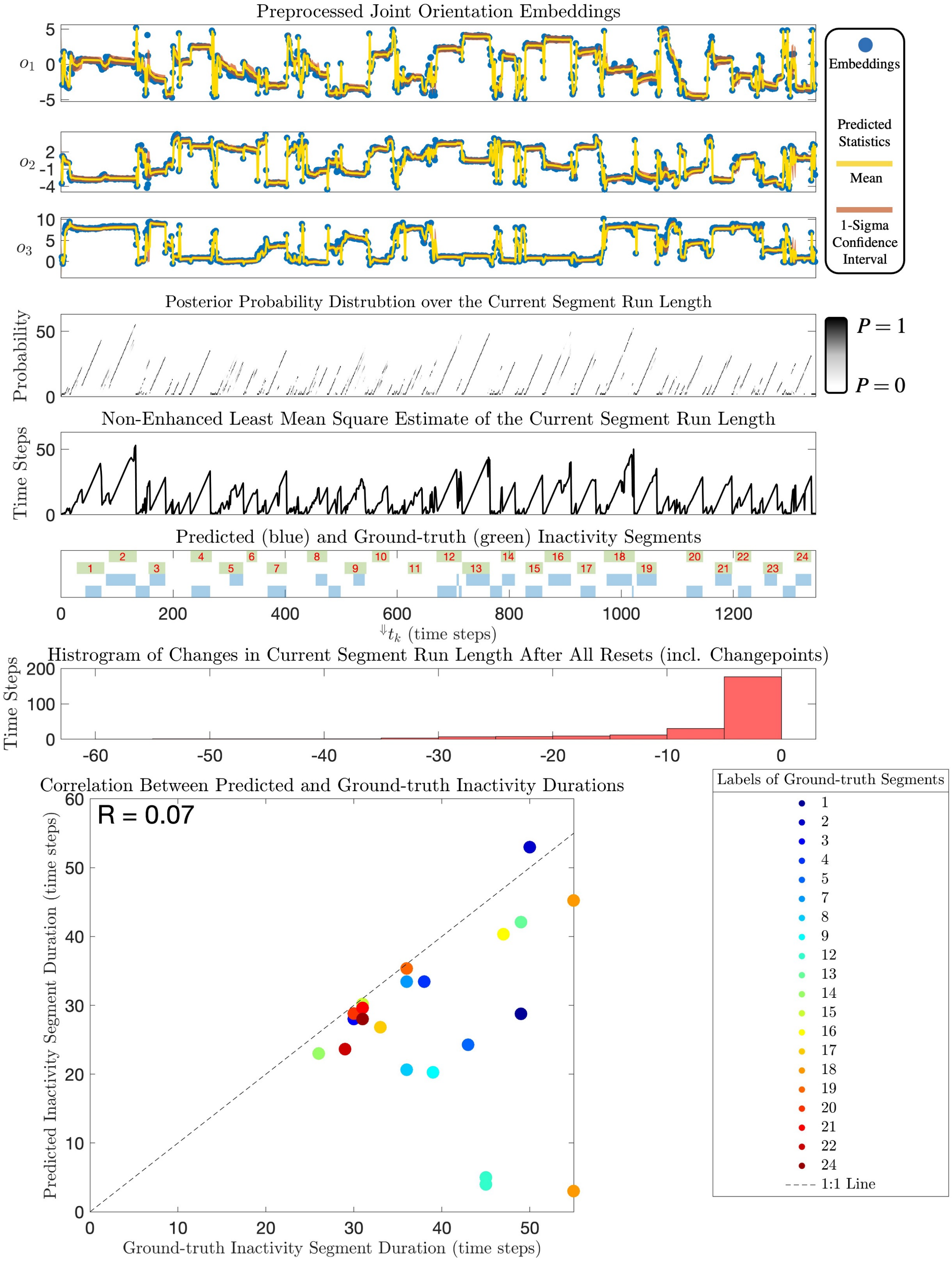

Figure S3. Performance visualisation for the framework variant, KIDS (I), applied to the dataset of participant (P1).

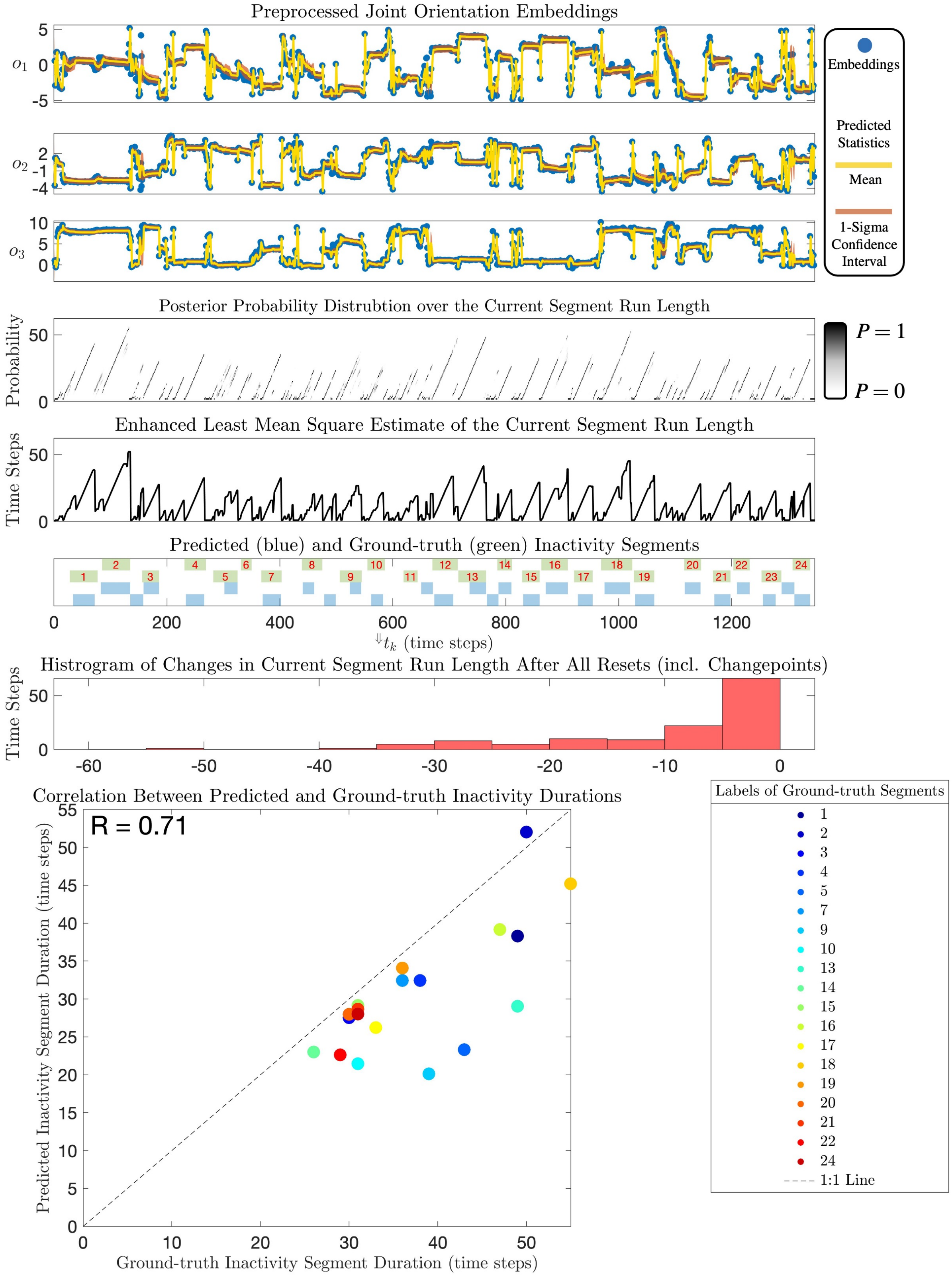

Figure S4. Performance visualisation for the framework variant, KIDS (II), applied to the dataset of participant (P1).

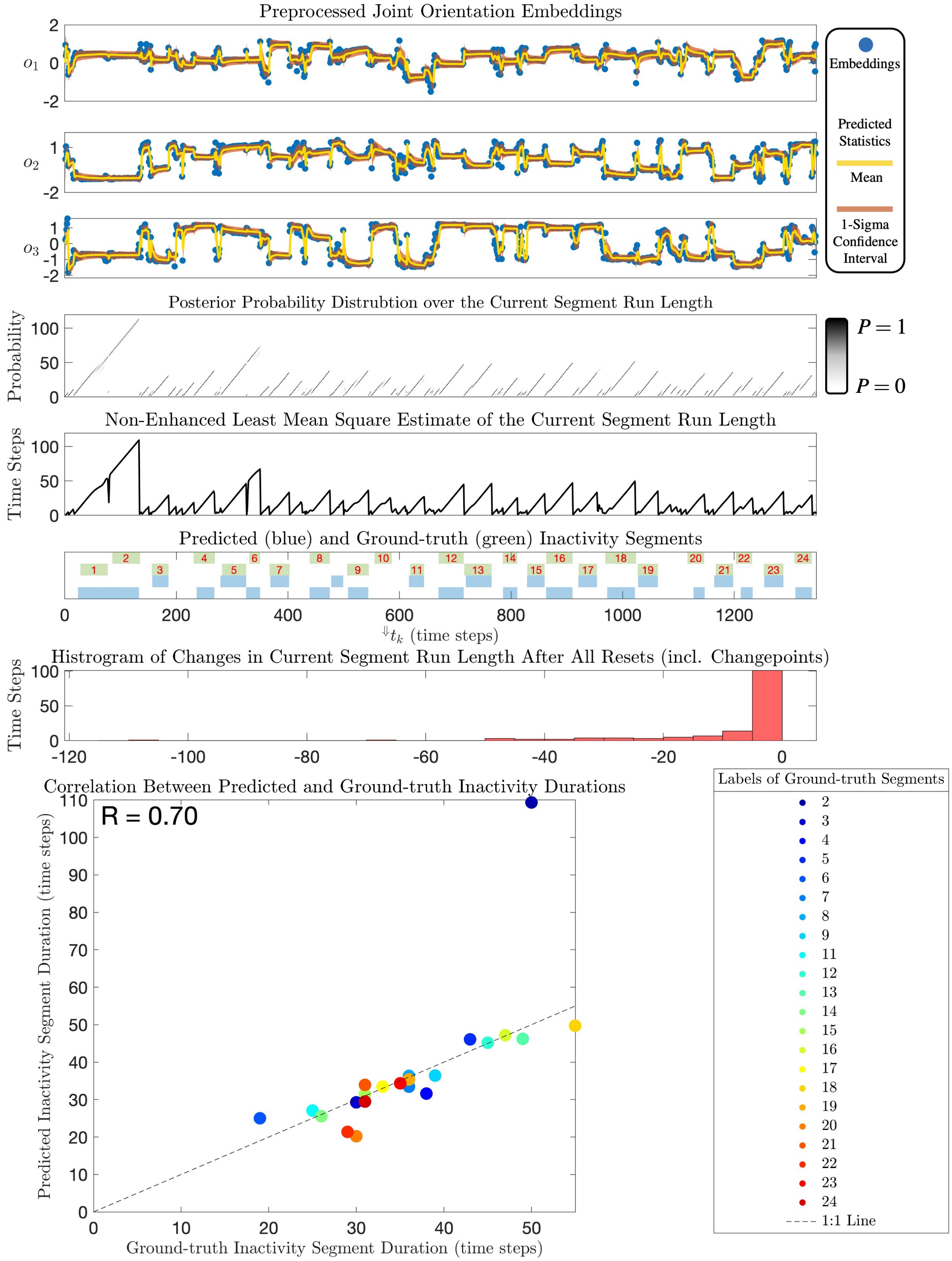

Figure S5. Performance visualisation for the framework variant, KIDS (III), applied to the dataset of participant (P1).

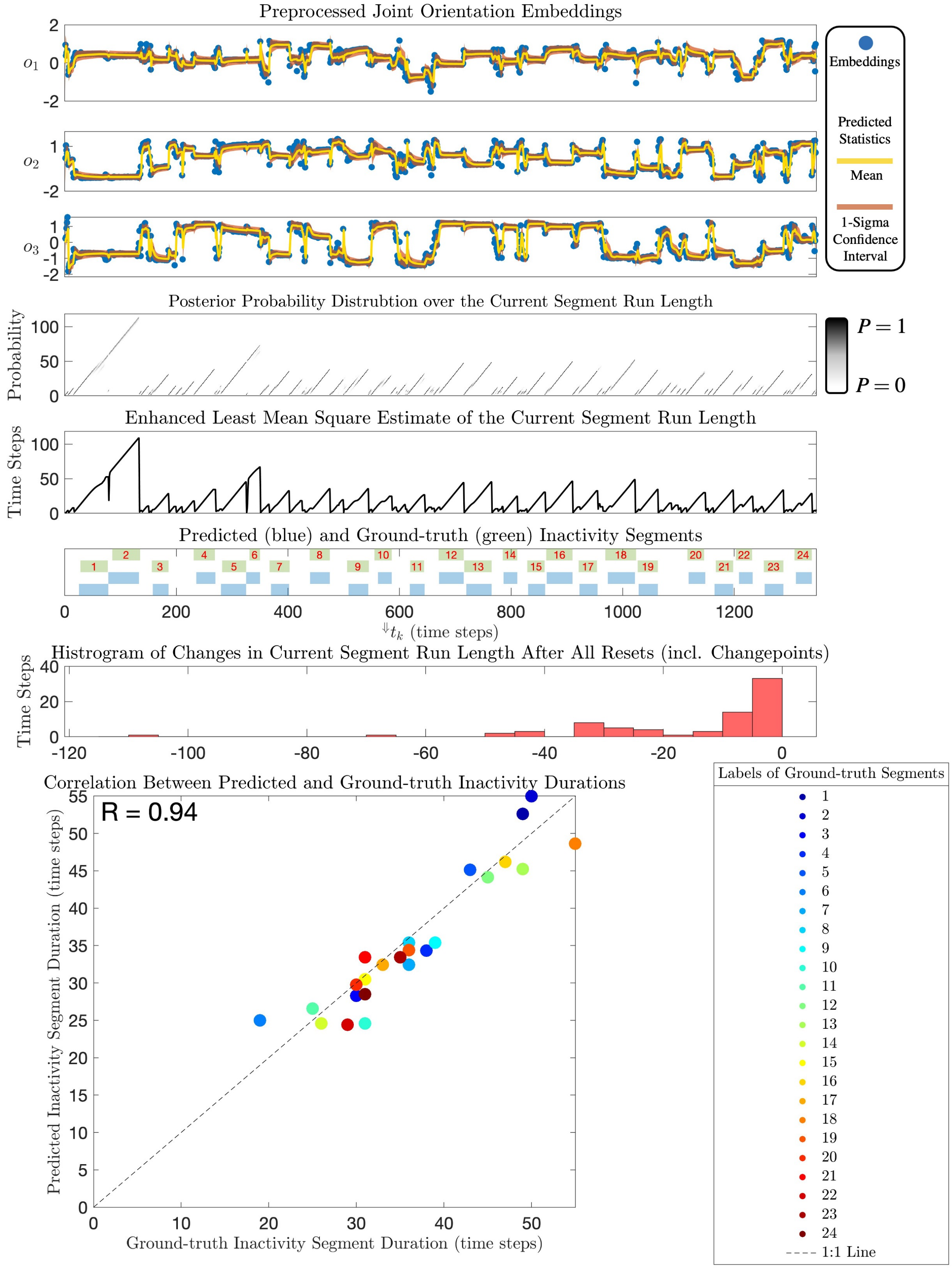

Figure S6. Performance visualisation for the framework variant, KIDS (IV), applied to the dataset of participant (P1).

Stage within KIDS Framework	Method Within Stage	Hyperparameter Description	Hyperparameter Value	Notes
Wrist Kinematics Measurement	Madgwick Filter	Orientation Adaptation Gain*	0.1	Controls the contribution of gyroscope measurements to the estimated orientation.
		Gyroscope Bias Compensation Gain*	0.015	Determines the rate at which the filter corrects for gyroscope bias.
Joint Kinematics Preprocessing and Visualisation	Uniform Manifold Approximation and Projection (UMAP)	Number of Nearest Neighbors*	199	Controls the balance between preserving local and global data structures.
		Minimum Distance between Points in the Embedding Space*	0.3	Controls the minimum distance between data points in the embedding space.
		Spread*	1	Controls the spread of embeddings.
		Distance Metric	Euclidean	Defines metric for point-to-point distance.
		Number of Epochs*	200	Number of iterations to refine embeddings.
	Analytical Dimension Reduction (ADR)	Inner Radius	1	Sphere's inner radius.
		Outer Radius	2	Sphere's outer radius.
Sleep Posture Change Detection<br>and<br>Temporal Segmentation of Postural Inactivity	Bayesian Inference	Mean Vector	$\begin{bmatrix}10^{-4} & 10^{-4} & 10^{-4}\end{bmatrix}^{\dagger}$<br>$\begin{bmatrix}10^{-4} & 10^{-4} & 10^{-4}\end{bmatrix}^{\bullet}$	A summary statistic for the Normal-Wishart prior.
		Covariance Scale Coefficient	10^{-4} † 0.05 •	A summary statistic for the Normal-Wishart prior.
		Number of Degrees of Freedom	4 † 4 •	A summary statistic for the Normal-Wishart prior.
		Scale Matrix	$\begin{bmatrix}625 & -500 & 125\\ -500 & 100 & -500\\ 125 & -500 & 625\end{bmatrix}^{\dagger}$<br>$\begin{bmatrix}5 & 0 & 0\\ 0 & 5 & 0\\ 0 & 0 & 5\end{bmatrix}^{\bullet}$	A summary statistic for the Normal-Wishart prior.
	Changepoint Detection Logic	Minimum Change in Current Segment Run Length	0.3	Minimum drop in the Log scale for the least mean square estimate of the Current Segment Run Length.
		Minimum Inactivity Segment Duration	20	Discards short-lasting inactivity segments during posture transitions.

Table S1. The hyperparameters employed within all stages of the proposed KIDS framework. The values of all hyperparameters labelled (*) were manually adjusted through a qualitative assessment of their respective method outputs based on either synthetically generated data or data from preliminary (unworn) sensor calibration procedures. The remaining hyperparameters were initialised purely based on a methodological design approach. Within the Bayesian inference method, the summary statistics of the Normal-Wishart prior were set differently depending on whether the prior was non-informative (†) or informative (•).
